# Single-cell transcriptomic analyses reveal cellular and molecular patterns of rose petal responses to gray mold infection

**DOI:** 10.1093/hr/uhaf152

**Published:** 2025-06-09

**Authors:** Xuejiao Li, Yinqi Siman, Yan Zhao, Lvchun Peng, Hongzhi Wu, Wenling Guan, Jingli Zhang, Yanfei Cai, Zhengan Yang, Gengyun Li, Jing Meng, Shuilian He

**Affiliations:** College of Landscape and Horticulture, Yunnan Agricultural University, Kunming, Yunnan 650201, China; College of Landscape and Horticulture, Yunnan Agricultural University, Kunming, Yunnan 650201, China; College of Landscape and Horticulture, Yunnan Agricultural University, Kunming, Yunnan 650201, China; Institute of Flowers, Yunnan Academy of Agricultural Sciences, Kunming, Yunnan 650201, China; College of Landscape and Horticulture, Yunnan Agricultural University, Kunming, Yunnan 650201, China; College of Landscape and Horticulture, Yunnan Agricultural University, Kunming, Yunnan 650201, China; College of Landscape and Horticulture, Yunnan Agricultural University, Kunming, Yunnan 650201, China; Institute of Flowers, Yunnan Academy of Agricultural Sciences, Kunming, Yunnan 650201, China; College of Landscape and Horticulture, Yunnan Agricultural University, Kunming, Yunnan 650201, China; Key Laboratory of Vegetable Biology of Yunnan Province, College of Landscape and Horticulture, Yunnan Agricultural University, Kunming, Yunnan 650201, China; College of Landscape and Horticulture, Yunnan Agricultural University, Kunming, Yunnan 650201, China; College of Landscape and Horticulture, Yunnan Agricultural University, Kunming, Yunnan 650201, China; College of Landscape and Horticulture, Yunnan Agricultural University, Kunming, Yunnan 650201, China; Key Laboratory of Vegetable Biology of Yunnan Province, College of Landscape and Horticulture, Yunnan Agricultural University, Kunming, Yunnan 650201, China

## Abstract

Roses *(Rosa hybrida*) are the most popular cut flower plants worldwide, accounting for over a third of the global cut flower industry. Gray mold, caused by *Botrytis cinerea*, is often referred to as the postharvest "cancer" of cut roses and represents the most significant disease impacting the postharvest preservation of these flowers in China. Currently, research progress in this area has been limited. Our study utilized single-cell RNA sequencing technology to elucidate the mechanisms underlying *B. cinerea* resistance in *R. hybrida* “Jumilia.” We identified seven distinct cell groups within rose petals. The rose epidermis acts as the physical barrier of defense against *B. cinerea*, while the infection rate may be accelerated through vascular tissues. Furthermore, we identified several key genes, including pectin methylesterases, pathogenesis-related proteins, glutathione S-transferase, and endochitinase EP3, which may play crucial roles in the stress response. The biosynthesis of secondary metabolites temporarily mitigates the infection process, and pathogenesis-related proteins have been recognized as key regulatory genes. This preliminary study elucidates the cellular changes and molecular mechanisms involved in *B. cinerea* infection in rose petals at the single-cell level. Our findings provide new insights into the defense mechanisms of roses against fungal diseases.

## Introduction

Roses (*Rosa hybrida*) are the most popular cut flower plants globally and represent more than a third of the global cut flower industry [1]. China ranks among the top three major producers of cut roses. However, fungal diseases significantly impact cut rose production, with gray mold being particularly severe. Gray mold is caused by *Botrytis cinerea*, a pathogen that is widely distributed in nature and able to affect more than 1400 plant species [2, 3]. The secondary metabolites produced by *B. cinerea* after germination can induce apoptosis in host cells, leading to necrosis of the plant tissues [2]. The development of gray mold is typically not apparent prior to the harvest of cut roses [4]. However, the senescence of cut rose petal cells following harvest, along with their unique conical structure, may facilitate rapid gray mold outbreaks [5]. Additionally, the deposition of pink pigment on the petals of light roses is associated with gray mold infection [6], and pigment patches on the petals of cut roses typically lead to a decline in quality grading. Sometimes referred to as the postharvest "cancer" of cut roses, gray mold is the most significant disease affecting the postharvest preservation of these flowers in China, with postharvest losses in Yunnan often exceeding 30% due to gray mold [4].

Plants employ complex strategies to resist pathogen invasion, including enhancing resistance through anthocyanin synthesis. Anthocyanin synthesis is typically induced when plants are subjected to abiotic stresses including drought, high salinity, excessive light, and cold [7], as well as to biological stresses from pests and diseases [8, 9]. This synthetic response is closely linked to a plant's capacity to withstand adversity. Under stressful conditions, the levels of reactive oxygen species (ROS) in plants increase. Anthocyanins are potent antioxidants and, trapping and neutralizing the ROS, effectively eliminate them, protecting the cells from oxidative damage and maintaining the oxidation–reduction equilibrium [10]. Moreover, anthocyanins also promote plant growth and development [10, 11]. In certain plants, the accumulation of anthocyanins is closely linked to enhanced disease resistance, enabling them to better withstand biological stresses such as fungi and bacteria, as well as insect pests [8, 9]. Various abiotic stress conditions can trigger the production of specific anthocyanins, suggesting that the biological roles of anthocyanins in plant stress responses are multifaceted. These roles may encompass ROS clearance, photoprotection, stress signal transmission, and the regulation of metabolic pathways [12]. During gray mold infection of roses, the petal color changes and the anthocyanin content increases; however, the precise role of anthocyanins in the infection process remains unclear.

Plant defense against disease has two primary stages. The initial immunity relies on cell surface immune receptors that detect pathogen-associated molecular patterns (PAMPs) and damage-associated molecular patterns (DAMPs) from and distinguish them from the molecular patterns of the hosts [13]. PAMPs play a crucial role in the resistance to *B. cinerea* [14]. The cell surface receptor-like protein encoded by the RBPG1 gene has been reported to recognize the endopolygalacturonase produced by *B. cinerea* [15]. Furthermore, the WAKs/WAKLs genes are known to encode proteins with a transmembrane structure, which features an extracellular domain containing a galacturonan binding site, which plays a crucial role in the detection of environmental stress [16–18]. However, knowledge of the plant cell surface immune receptors that confer resistance to *B. cinerea* remains insufficient, with only a limited number of receptor genes reported.

Transcription factors play an important role in responding to the stress of *B. cinerea* infection. Infection with *B. cinerea* has been shown to upregulate the expression of 19 *RcWRKYs* [19]. Of these, *RcWAK4* has been identified as being involved in the defense of roses against *B. cinerea* [16]. Analysis of the MYB transcription factor family revealed that *RcMYB84* and *RcMYB123* mediate the jasmonic acid pathway, which was able to induce the rose defense response against *B. cinerea* [20]. Additionally, five genes (*RcbHLH13*, *RcbHLH35*, *RcbHLH41*, *RcbHLH44*, and *RcbHLH49*) from the *bHLH* gene family have been identified as being involved in rose resistance to *B. cinerea* [21].

Single-cell RNA sequencing (ScRNA-seq) enables the acquisition of transcriptome expression profiles from millions of individual cells at various developmental stages, allowing for the extraction of genetic information at the single-cell level. It reveals the gene structure and expression status of each cell, accurately reflecting inter-cell heterogeneity and facilitating the inference of developmental trajectories of cell types across different time periods [22, 23]. scRNA-seq was initially applied to the study of human and animal diseases [24, 25]; however, in recent years, its application has expanded to investigations concerning plant growth, development, and resistance. For instance, scRNA-seq has been used to elucidate the transcriptional regulation and response to heat stress during leaf development in *Brassica rapa* [26]. Additionally, it has been employed to create the first single-cell map of the leaves of the medicinal plant *Catharanthus roseus* (Madagascar periwinkle), revealing the spatial distribution of the vinblastine biosynthesis pathway within the leaves [27]. The preparation of plant single-cell suspensions is inherently more complex than that of animal cell suspensions, as it necessitates the digestion of the cell wall. Despite this complexity, there are limited reports on the application of single-cell transcriptome sequencing technology for investigating the response of plants to disease stress, particularly in roses, which hold significant economic value.

*R. hybrida* “Jumilia” is a fast-growing and elegant cut rose variety that exhibits high susceptibility to gray mold. To elucidate the mechanisms underlying *B. cinerea* resistance in *R. hybrida*, we measured phenotypic and physiological data from petals at various stages of gray mold infection. Subsequently, scRNA-Seq was conducted on protoplasts isolated from “Jumilia” petals at different stages of infection with *B. cinerea*, allowing us to define distinct cell clusters based on cell-to-cell variation in gene expression. Our gene co-expression analysis using scRNA-Seq datasets, in conjunction with *in vivo* characterization of enzyme activity, enhances our understanding of the pathogenic mechanisms of gray mold in *R. hybrida*.

## Results

### Single-cell transcriptome sequencing and gene expression profile of rose petals

**Figure 1:**
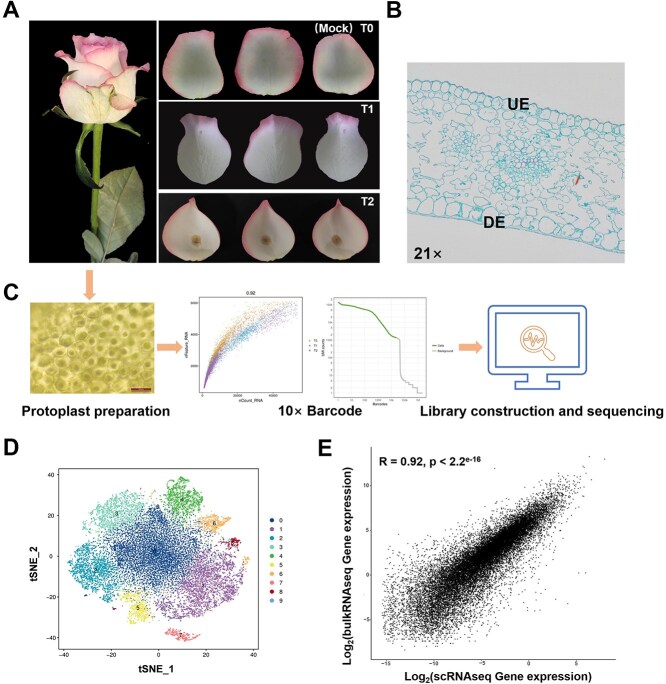
Single-cell transcriptome sequencing analysis of *R. hybrida* petals. **(A)** Flower petals of the variety *R. hybrida* “Jumilia” used in this study. T0(mock): uninfected petals; T1: early stage of infection with *B. cinerea*, with red spots appearing on petals; T2: intermediate stage of infection, with a large area of brown spots appearing on the petals. **(B)** Paraffin structure diagram of uninfected rose petals in longitudinal section. UE represents upper epidermis, DE represents lower epidermis. **(C)** Preparation of protoplasts, single-cell isolation, library construction, and high-throughput sequencing of rose petals. **(D)** The t-distributed stochastic neighbor embedding (tSNE) plot of rose petal cell subpopulations. The data were analyzed and the samples were divided into 10 cell subpopulations. **(E)** Correlation coefficients of bulkRNAseq and scRNAseq in the samples (*R* = 0.92).

Petals are the primary organs affected by *B. cinerea* in roses. To elucidate the mechanism of *B. cinerea* infection at the cellular and gene expression levels, we selected petals from the highly susceptible *R. hybrida* “Jumilia” for infection experiments. Petals at three stages of infection were chosen for single-cell sequencing: those that were not infected as mock (T0), those at the early stage of infection (T1), and those at the intermediate stage of infection (T2) (Fig. 1A). At the same time, we also performed morphological and anatomical observations on the petals at these three stages of infection (Fig. 1B). A total of 449 378 442; 455 297 969, and 407 805 682 reads were obtained from samples at T0, T1, and T2, respectively. Of these, valid barcodes accounted for 97.10%, 95.90%, and 97.30% of the total reads, respectively. We analyzed 9189 (T0), 9185 (T1), and 6220 (T2) high-quality cells, and the corresponding gene annotations were 27 959, 26 648, and 26 649. The average percentage of reads confidently mapped to the genome was 86.7%, while the average Q30 bases in unique molecular identifiers (UMI) across the three samples was 97.0% (Fig. 1C, Table S1).

Based on the classification of cell subpopulations, we employed the *t*-distributed stochastic neighbor embedding (tSNE) nonlinear clustering method to visualize the identified single-cell subpopulations, and the samples were then categorized into 10 distinct subpopulations (Fig. 1D). Additionally, we examined the correlation between bulk RNA sequencing (bulkRNAseq) and scRNAseq results under identical conditions. The Spearman correlation coefficient was found to be 0.92, indicating a high degree of consistency between the two sets of results (Fig. 1E).

### Identification of the main cell clusters in rose petals

**Figure 2:**
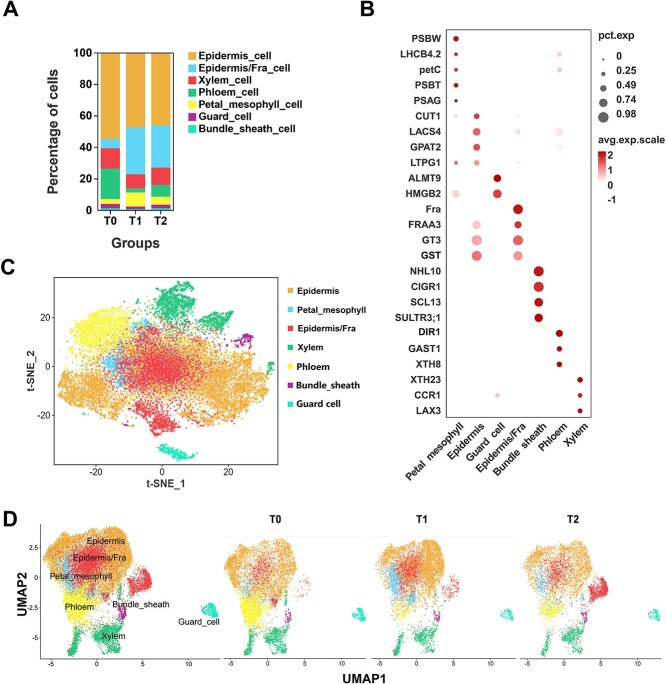
Identification of subpopulations of rose petal cells. **(A)** Cell counts for the 7 cell subpopulations identified based on marker genes in cells at each of the three tested stages of infection. **(B)** Markers used for cell identification in rose petals. **(C)** The t-SNE maps of the cell subgroups identified in rose petals. **(D)** UMAP maps of cell subgroups in the identified petals at infection stages T0, T1, and T2. Changes in cell number among different subgroups in the samples at each infection stage are shown.

After multicellular complexes and abnormal cells had been eliminated, we obtained a total of 8390, 8522, and 5840 cells at stages T0, T1, and T2, respectively (Table S2). To date, there have been few single-cell reports focusing on petals. To enhance the identification of cell types within rose petals, we referenced marker genes identified in other species to facilitate a comprehensive identification of cells in our study [27–31]. Based on the sequencing results, we categorized the rose petal cells into seven subgroups. The NDR1/HIN1-like protein 10 (NHL10) and scarecrow-like protein 21 (CIGR1) were identified as bundle sheath cell marker genes. Glycerol-3-phosphate acyltransferase 2 (GPAT2), 3-ketoacyl-CoA synthase 6 (CUT1), and long chain acyl-CoA synthetase 4 (LACS4) are epidermal marker genes. Aluminum activated malate transporter 9 (ALMT9), HMG1/2-like protein, and transcript variant X1 (HMGB2) were identified as guard cell marker genes. Chlorophyll a-b binding protein CP29.2 (LHCB4.2), photosystem II 5 kDa protein (PSBT), and photosystem II reaction center W protein (PSBW) were classified as petal mesophyll marker genes. The protein GAST1 (GAST1), xyloglucan endotransglucosylase/hydrolase protein 8 (XTH8), and lipid-transfer protein DIR1 were designated as phloem marker genes. Cinnamyl CoA reductase 1 (CCR1), xyloglucan endoglucosidase/hydrolase protein 23 (XTH23), and auxin transporter-like protein 3 (LAX3) are components of xylem cells. Notably, in contrast to previous reports, the major strawberry allergen Fra 1.08 (Fra), which is associated with pigment formation, was found to be significantly overexpressed in cluster 4, and they were significantly expressed in the subgroup of epidermal cells. Consequently, we classify the cells in this subgroup as epidermal/Fra cells. Utilizing this marker gene information, we identified seven distinct cell types: epidermis cells (EC), epidermis/Fra cells (EC/Fra), xylem cells (XC), phloem cells (PHC), petal mesophyll cells (PMC), guard cells (GC), and bundle sheath cells (BSC) (Fig. 2A and B; Table S3).

### Single-cell expression profile analysis of rose petals infected with *B. cinerea*

A total of 8390 cells were identified in uninfected petals: 4574 epidermal cells, 500 epidermis/Fra cells, 1093 xylem cells, 1627 phloem cells, 254 petal mesophyll cells, 229 guard cells, and 113 bundle sheath cells. During the early stage of infection, we obtained a total of 8522 cells, including 4020 epidermal cells, 2551 epidermis/Fra cells, 745 xylem cells, 238 phloem cells, 764 petal mesophyll cells, 125 guard cells, and 79 bundle sheath cells. A total of 5840 cells were obtained from petals at the intermediate stage of infection, comprising 2689 epidermal cells, 1565 epidermis/Fra cells, 639 xylem cells, 440 phloem cells, 295 petal mesophyll cells, 133 guard cells, and 79 bundle sheath cells (Fig. 2A, Table S2). We observed that during the intermediate stage, the number of cells in the petal significantly decreased, particularly epidermal, phloem, and xylem cells. Conversely, the number of epidermis/Fra cells significantly increased in both the early and intermediate stages of infection, as confirmed by the visualization results from the uniform manifold approximation and projection (UMAP) of three samples (Fig. 2D).

### Analysis of significantly differentially expressed genes in cell subpopulations

**Figure 3:**
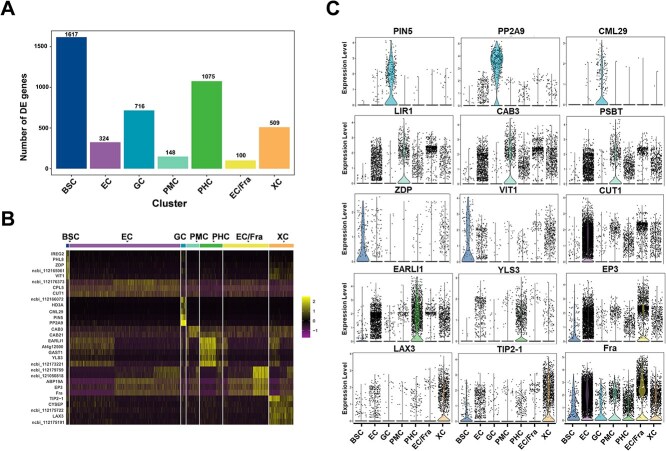
Significant differentially expressed genes analysis in different types of rose petal cells. **(A)** The number of up-regulated DEGs in different cell subpopulations. The screening criteria were log_2_FC ≥ 0.36 and *P*-value ≤0.01, and the genes must be expressed in more than 25% of cells in the target subgroup. **(B)** Heatmap of the top five significantly up-regulated DEGs in each subpopulation. **(C)** Violin plot of partially up-regulated DEGs in the subpopulations. BCS, bundle sheath cells; EC, epidermis cells; GC, guard cells; PMC, petal mesophyll cells; PHC, phloem cells; EC/Fra, epidermis/Fra cells; XC, xylem cells.

A total of 1617 significantly differentially expressed genes (DEGs) were identified in BSC, representing the highest number of DEGs. The second highest number of DEGs was found in PHC (1075). EC, GC, PMC, EC/Fra, and XC were found to contain 324, 716, 148, 100, and 509 DEGs, respectively (Fig. 3A). Up-regulated genes in different subgroups were show in Supplementary Table S4. The top five significantly up-regulated DEGs in PHC, PMC, GC, and EC/Fra were screened and are presented in Fig. 3B. KEGG pathway analysis indicated that most of these genes were associated with metabolic pathways, plant hormone signal transduction, and plant–pathogen interactions (Fig. S1). Notably, CAB3 was linked to metabolic pathways, CUT1 was associated with the biosynthesis of secondary metabolites, plant–pathogen interactions, and fatty acid elongation. LAX3 was related to plant hormone signal transduction, while CML29 was connected to plant–pathogen interactions.

Most genes, including PIN5, PP2A9, and CML29, were significantly expressed in GC. LIR1, CAB3, and PSBT exhibited high expression levels primarily in PMC. ZDP and VIT1 were significantly expressed in BSC, while CUT1 was predominantly expressed in EC. EARLI1 and YLS3 were mainly expressed in PHC, and LAX3 and TIP2-1 were primarily found in XC. Interestingly, Fra was expressed across all subgroups, with the highest expression levels observed in EC/Fra (Fig. 3C, Table S5). In conjunction with bulk RNA sequencing, several genes were selected for qRT-PCR validation, the results demonstrated good consistency between techniques (Fig. S2).

### DEGs analysis of epidermal cells in rose petals

**Figure 4:**
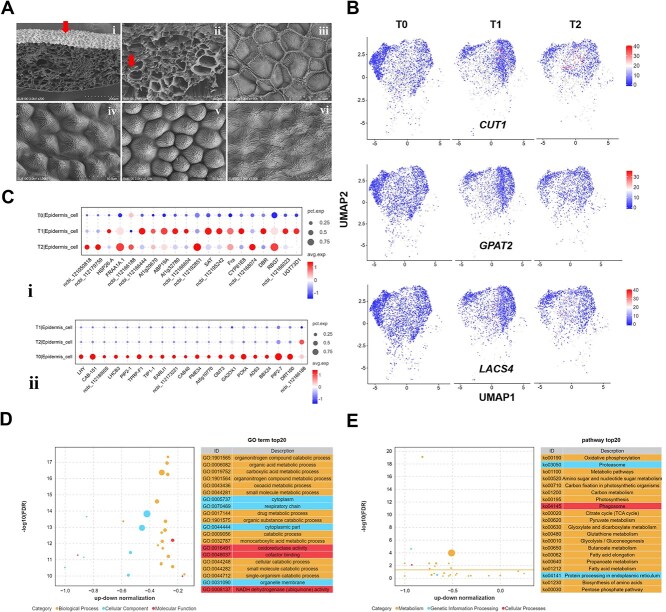
Morphology of rose petal cells on infection with *B. cinerea,* and differential gene expression profile in rose petal epidermal cells. **(A)** Scanning electron micrographs of rose petal cells. **i,** longitudinal section of the petals. The red arrow indicates the upper epidermis. **ii,** vascular tissue of the petals, with the vascular site indicated by the red arrow. **iii,** lower epidermis of rose petals. **iv**, **v** and **vi** show the changes in the upper epidermis of rose petal cells in stages T0, T1, and T2 of infection with *B. cinerea*, respectively. **iv,** normal morphology of the upper epidermis in uninfected rose petals. **v,** obviously swollen upper epidermis cells in the early stage of infection. **vi,** ruptured upper epidermis cells in the intermediate stage of infection. **(B)** UMAP of partial epidermal marker genes in rose petals. **(C)** Top 20 significantly up-regulated (**i**) and significantly down-regulated (**ii**) DEGs in epidermal cells. **(D)** GO terms analysis of epidermal cell DEGs in rose petal cells at three stages of infection. **(E)** KEGG pathway analysis of DEGs in different rose petal epidermal cells at three stages of infection with *B. cinerea*.

Mature petals typically consist of an adaxial epidermal layer, several layers of mesophyll cells, and an abaxial epidermal layer [32]. The physical structures of petals can, however, be intricate, often featuring elaborate characteristics such as lobes, fringes, nectary spurs, or hair pads [33]. Furthermore, even within a single flower, the petals are not uniform [34, 35]. The epidermis is the primary tissue in rose petals that enables them to resist adverse environmental stimuli. In this study, according to scanning electron microscope, we observed the changes occurring in the epidermis of rose petals during infection with *B. cinerea*. The upper epidermis, which comprises numerous conical cells, exhibited significant alterations on initial infection. Under uninfected conditions, these conical cells maintained a normal relaxed state (Fig. 4Aiv). However, during the early stage of infection (T1, Fig. 4Av), we observed that the conical cells swelled significantly, it is speculated that in response to unfavorable environmental stimuli. In the intermediate stage of infection (T2), the upper epidermis became noticeably wrinkled, indicating a state of dehydration, the epidermis showed signs of damage, and the number of epidermal cells are significantly reduced (Fig. 4Avi, Table S2).

However, the changes observed in the lower epidermis were not significant (Fig. 4Aiii). In this study, we found that the expression levels of the EC marker genes CUT1, GPAT2, and LACS4 were significantly reduced in most epidermal cells during the infection process, particularly at the T2 stage. Conversely, some epidermal cells exhibited a significant up-regulation of expression (Fig. 4B). To further elucidate the differential expression of EC genes, we analyzed the top 20 genes that were significantly up-regulated and down-regulated (*P* value = 0, FDR = 0) (Table S6). The results indicated that of the 20 up-regulated genes, FRAA1A, RBG7, ncbi_121050818, ncbi_112192851, ncbi_112179759, and ncbi_112168074 continue to exhibit increased expression throughout the infection process. In contrast, other genes were significantly up-regulated during the T1 stage but down-regulated in the T2 stage. Additionally, 20 genes, including CAB-151, LHCB3, PIP2–1, TIP1–1, EARLI1, CAB40, GA2OX1, and PIP2–7, were significantly down-regulated in both the T1 and T2 stages (Fig. 4C).

We next conducted GO and KEGG enrichment analysis on DEGs between different groups of epidermal cells. GO analysis mainly showed enrichment in two categories: biological processes and cellular components, which were mostly involved in the organonitrogen compound catabolic process (GO: 1901565), organic acid metabolic process (GO: 0006082), carboxylic acid metabolic process (GO: 0019752), and the organonitrogen compound metabolic process (GO: 1901564). KEGG pathway analysis revealed that the DEGs were enriched in pathways including oxidative phosphorylation (ko00190), proteasome (ko03050), and metabolic pathways (ko01100) (Fig. 4D and E). However, the up-regulated genes in rose epidermal cells were significantly enriched in pathways such as protein processing in the endothelial reticulum (ko04141), glycolipid metabolism (ko00561), fatty acid elongation (ko00062), and plant–pathogen interactions (ko04626) (Fig. S3A).

### Pseudotime analysis and changes in gene expression in rose petal epidermis/Fra cells under *B. cinerea* infection

During the early stage of infection (T1), transient red spots appeared on the affected area of the petals, which also exhibit the highest anthocyanin concentrations. Other related physiological indices were also significantly increased, with the anthocyanin and delphinidin concentrations peaking at the T1 stage. This observation was consistent with the phenotype observed during the same period (Fig. S4A). Subsequently, in stage T2, the petals turned brown, eventually drying out and becoming mildewy. In our transcriptome analysis, we found that genes associated with the flavonoid synthesis pathway were significantly upregulated during the intermediate stage of gray mold infection (Fig. S4B). We speculated that pigments, which are significant secondary metabolites in rose petals, initiate a defense response to the stress caused by *B. cinerea* infection. Consequently, we analyzed the developmental trajectory of epidermis/Fra cells, revealing three distinct differentiation trajectories and states within the samples.

**Figure 5:**
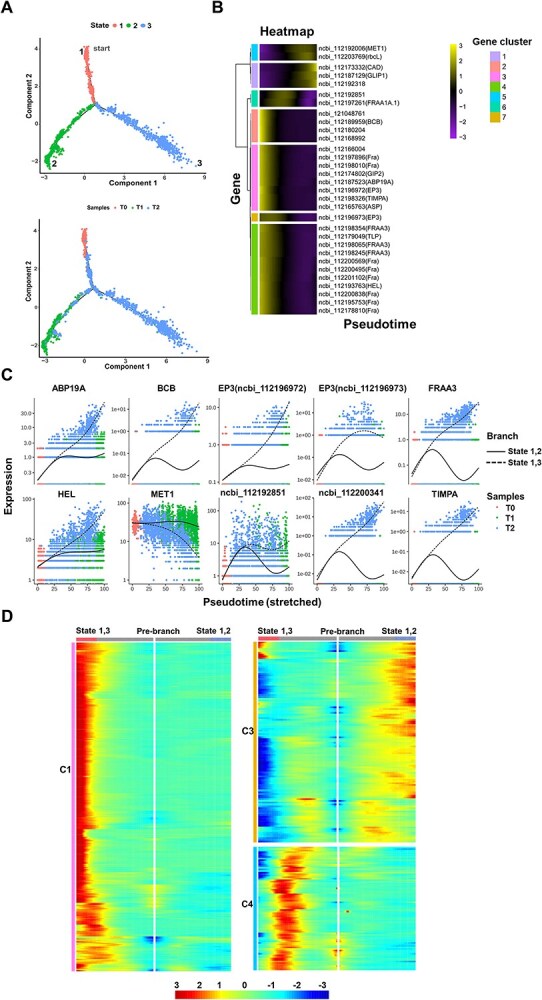
Pseudotime analysis and heatmap of DEGs in rose petal epidermis/Fra cells. **(A**) The pseudotime differentiation trajectories of epidermis/Fra cells and the distribution of the three stages of samples in the cell trajectory, epidermis/Fra cells were divided into three differentiation states, with state 1 designated as the starting point of cell differentiation. The pseudotime differentiation states corresponded well with the distribution of the three stages of samples in the cell trajectory. **(B)** Heatmap of significantly DEGs in three differentiation states (*P* value = 0, FDR = 0), where “gene cluster” represents a class of genes with similar expression trends. **(C)** The trajectories of the top 10 significantly DEGs with distinct fates over pseudotime were identified. The *x*-axis represents the pseudotime point, the *y*-axis represents the gene expression level, the black smoothed and dotted lines represent the fitted lines of gene expression levels in different branches, and different colors represent samples at different stages of infection. **(D)** DEGs during the process of epidermis/Fra cell differentiation. These genes were divided into 5 clusters. Genes in clusters C1, C3, and C4 showed specific expression trends during the differentiation process. The genes in C2 and C5 were mainly expressed in the early stages of the differentiation process and are shown in Supplementary Fig. S5. The horizontal axis represents the pseudotime point, which gradually increases from the middle towards both sides, and the vertical axis represents the gene expression level. The left and right sides each have a branch, and clusters represent the gene sets with similar branch expression trends, with different colors representing high or low levels of expression.

We plotted the developmental trajectories of epidermis/Fra cells at various stages of infection to elucidate the differentiation process of epidermis/Fra cells during gray mold infection. Epidermis/Fra cells were categorized into three differentiation trajectories based on their differentiation states (Figs 5A and S5). In this analysis, state 1 was designated as the initial point of differentiation. We found that the cell distribution of the samples across the three stages of infection corresponded strongly with the three differentiation states. A total of 500, 2,551, and 1,565 epidermis/Fra cells were screened in the T0, T1, and T2 stages, respectively. Of these, 802 cells were identified in state 1, which included 496 cells from the T0 stage, representing 99.2% of the epidermis/Fra cells in the T0 sample. We counted 2,711 cells in state 2, which included 2,542 cells from the T1 stage, accounting for 99.65% of the epidermis/Fra cells in the T1 sample. In state 3, 1,103 cells were identified, with 1,096 cells in the T2 stage, accounting for 70.03% of the epidermis/Fra cells in the T2 sample. During this process, we identified 31 DEGs (*P*-value = 0, FDR = 0) across these three states. The expression heatmap indicated that most genes were highly expressed during the early stage of differentiation, while FRAA1A.1 and ncbi_112192851 exhibited high expression levels in the intermediate stage of differentiation, suggesting their active participation in the cell differentiation process (Fig. 5B). DEGs with distinct fates over pseudotime were identified, and a trajectory graph illustrating the expression levels of the top 10 significantly DEGs was generated. DEGs between states 1 and 2 were primarily up-regulated before being subsequently down-regulated, whereas those between states 1 and 3 were predominantly up-regulated (Fig. 5C). We observed that ABP19A, BCB, FRAA3 (ncbi_112198065, ncbi_112198245), HEL, ncbi_112200341, and TIMPA were significantly upregulated, and had particularly elevated levels during the T2 stage. In contrast, TPRP-F1, RCI2A, and XTH6 exhibited continuous downregulation.

Based on these expression trends, genes exhibiting differential fates in epidermis/Fra cell differentiation were categorized into five clusters (Figs 5D and S5B). Notably, during the differentiation processes, a significant number of genes in clusters 1 (C1) and 4 (C4) were found to be highly expressed during the mid- and late- stages of differentiation. This suggests that genes in C1 and C4 may play an active role in the differentiation of epidermis/Fra cells. In contrast, genes in cluster 3 (C3) showed high expression levels primarily during the late stage of the differentiation processes in states 1 and 2. Expression of genes in clusters C1 and C4 was more significantly associated with the differentiation process, and these genes were mainly related to biosynthesis of secondary metabolites such as phenylpropanoid biosynthesis (ko00940), alkaloid biosynthesis (ko00960), and flavonoid biosynthesis (ko00941). The genes in C2 and C5 were mainly expressed in the early stages of the differentiation process (Fig. S5B, Table S7). Furthermore, DEGs were screened based on the pseudotime values of each cell, and the expression trends over pseudotime were plotted for the top 10 significantly DEGs (Fig. S5C).

### Analysis of disease-related genes in rose petals

**Figure 6:**
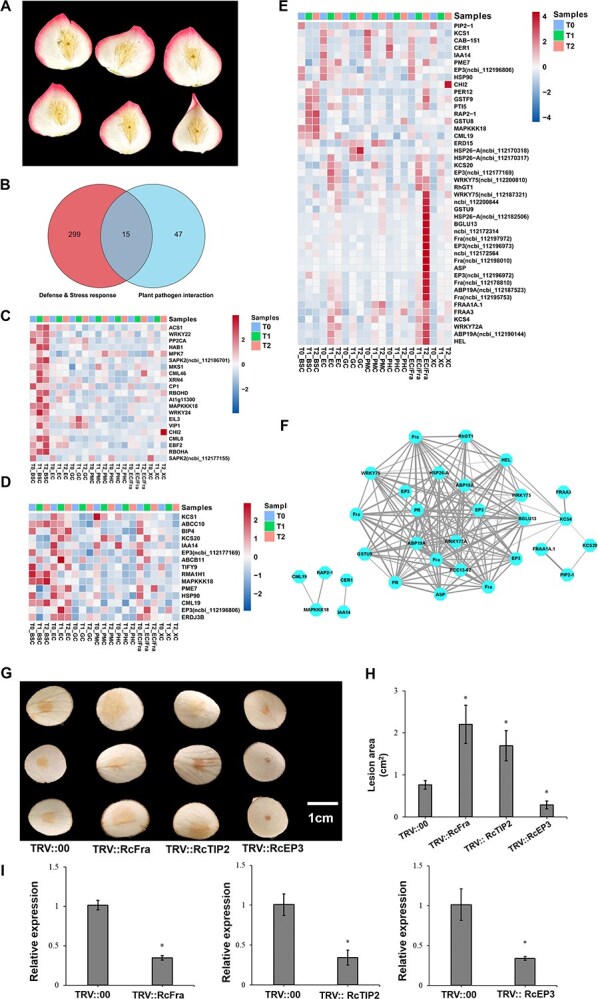
Progression of *B. cinerea* infection in rose petals and the identification of disease-related genes. **(A)** The morphological changes in rose petals infected by *B. cinerea.* After initial infection, the fungi spread rapidly through the vascular tissues. **(B)** Venn diagram of DEGs in petal mesophyll cells based on significant GO enrichment pathways, revealing 15 DEGs in two pathways. **(C)** Heatmap of the 15 significantly DEGs in **B**. **(D)** Heatmap of the MAPK signaling pathway DEGs in bundle sheath cells. **(E)** Heatmap of expression of genes with continuously up- or down-regulated expression identified in the trend analysis. **(F)** Network regulatory relationships among the focus genes based on trend analysis (correlation coefficient 0.9, *P* < 0.05). **(G)** Disease symptoms of TRV::00, TRV::RcFra, TRV::RcTIP2 and TRV::RcEP3 petals infected by *B. cinerea* for 72 hours. **(H)** Lesion area of TRV::00, TRV::RcFra, TRV::RcTIP2 and TRV::RcEP3 petals infected by *B. cinerea* for 72 hours. The average lesion size from three biological replicates. Significant differences are presented as means ± SD of three biological replicates (Student’s *t* test, *P < 0.05). **(I)** The relative expression level of TRV::00, TRV::RcFra, TRV::RcTIP2 and TRV::RcEP3 petals infected by *B. cinerea* for 72 hours.

During the intermediate stage of *B. cinerea* infection in roses, disease spots radiate outward from the initially infected area. The petal mesophyll and vascular tissue exhibit yellow-brown spots that progressively expand. The color of the vascular tissue deepens initially, suggesting that the infection may have expedited the infection process through the vascular tissue, serving as the primary route of infection (Fig. 6A). Combined GO and KEGG analyses revealed that the most significant terms in petal mesophyll cells were the defense response (GO: 0006952) and response to stress (GO: 0006950) (Fig. S6). We constructed Venn diagrams based on the genes enriched in these two pathways, as well as in the plant–pathogen interaction pathway, resulting in a total of 15 significantly DEGs (Fig. 6B). The heatmap indicated that these genes, including MAPKKK18, HSP90, EP3, ABCC10, and CML19, were significantly overexpressed in both BSC and EC (Fig. 6C). Meanwhile, the MAPK signaling pathway (ko04016) was found to be significantly enriched in BSC, with genes associated with this pathway, including WRKY22, MPK7, PP2CA, MAPKKK18, WRKY24, BIL3, VIP1, and CML8, closely linked to pathogen infection (Fig. 6D). Additionally, we performed a trend analysis on the gene expression across samples taken from three stages of infection, categorizing the expression responses into eight distinct expression trends. Profiles 0, 1, and 6 exhibited the most significant differences in expression patterns (Fig. S7). The significantly up-regulated and down-regulated genes across the three trends were screened, resulting in the identification of 44 pathogenesis-related and defense-related genes for the construction of an expression heatmap (Fig. 6E). From the figure, it was evident that these genes were predominantly expressed in BSC, EC, and EC/Fra cells, with the most pronounced expression observed in the EC/Fra cells at infection state T2. Additionally, complex regulatory network relationships were identified among Fra, EP3, HSP26-A, PR, ASP, BGLU13, WRKY75, KSC4, and ABP19A (Fig. 6F).

Three potential candidate genes *RcFra*, *RcTIP2*, and *RcEP3* were selected for transient silencing expression experiment in rose petals. Compared with the control (TRV—control) in “Jumilia” rose petals, it was observed that the lesion areas of *RcFra*-silenced and *RcTIP2*-silenced increased significantly after inoculated with *B. cinerea* at 72 hours, whereas that of *RcEP3*-silenced decreased significantly (Fig. 6G–I). These results suggest that *RcFra* and *RcTIP2* may positively regulate the stress response to against *B. cinerea*, while *RcEP3* plays a negative regulatory role.

**Figure 7:**
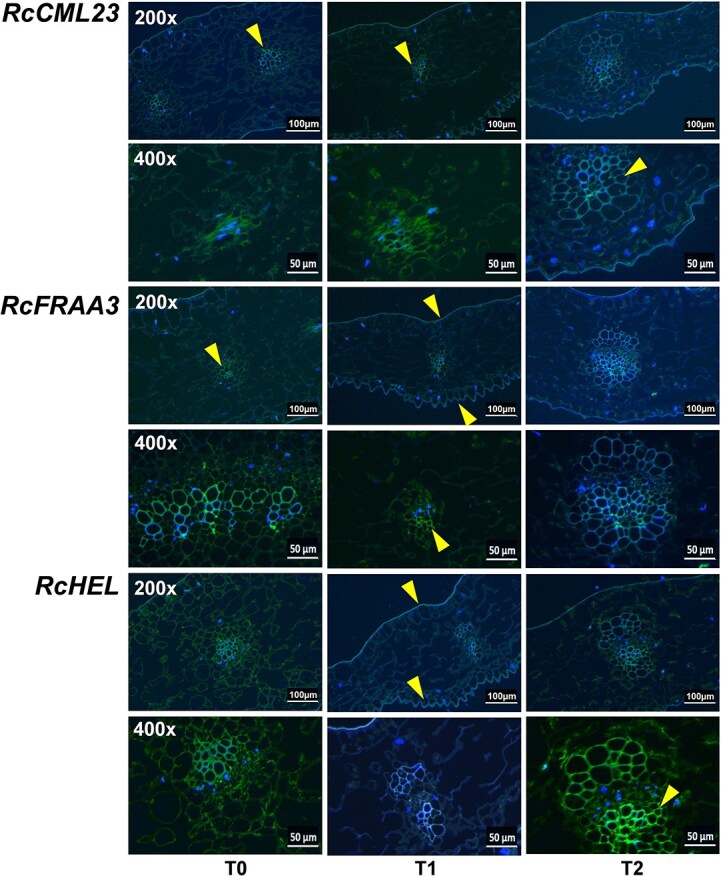
*In situ* hybridization of significantly DEGs during the *B. cinerea* infection process in rose petals. Green fluorescence indicates the expression of the target gene, and yellow arrows indicate the expression site of the target gene in rose petals.

Three DEGs were further analyzed through *in situ* hybridization experiments. The calcium-binding protein *RcCML23* was expressed prominently in the BSC, whereas the major strawberry allergen Fra a 1–3 (*RcFRAA3*) and the pathogenesis-related protein PR-4 (*RcHEL*) were found within the epidermis/Fra cells, and showed significant expression in the upper and lower epidermis as well as in the vascular tissue of the petals (Fig. 7).

## Discussion

### Changes in the morphology of rose petals following *B. cinerea* infection

*B. cinerea* is a major disease affecting roses throughout the cultivation and harvesting processes. In Yunnan, China, which is a major production area for cut roses, the warm and rainy conditions during summer create a high risk for *B. cinerea* infection. In facility cultivation, it is necessary to spray crops with fungicides weekly as a precaution against infection, resulting in extremely high financial and environmental costs. During the early stages of infection in facility-grown roses, red spots appear on the petals. These spots can persist for a week or longer, but subsequently, gradually turn gray and develop a mold layer. *B. cinerea* typically undergoes a brief asymptomatic nutritional stage in the early phases of disease development [36]. Previous studies have demonstrated that *B. cinerea* infection is a multi-layered process controlled by the interaction of various factors, which collectively influence the severity and overall progression of the disease [36–38]. Rose petals may experience a dynamic equilibrium between autophagy (AU) and apoptosis (AP) during *B. cinerea* infection. Autophagic cell death enhances plant resistance, whereas the inhibition of autophagy allows fungi to grow and accumulate biomass within host tissues [36]. Once a sufficient critical mass has been achieved, the fungi produce compounds that trigger apoptosis, marking the onset of cell death and necrotic tissue formation. The observed phenotype suggests that the appearance of red spots represents a critical period for roses to manage infection stress, with significant differences noted between living and cut-flower. This distinction may serve as a pivotal breakthrough point for developing potential strategies to manage this disease.

### The identification of cell subpopulations in rose petals

Mature petals typically consist of an adaxial epidermal layer, mesophyll cells, and an abaxial epidermal layer. The epidermal cells exhibit distinct differentiation characteristics, particularly in the presence of typical papillary conical cells. These conical cells represent significant cellular innovations in flowering plants, enhancing the color intensity of petals and imparting a shiny appearance that attracts pollinators, and influencing the overall shape of the petals [39–43].

In this study, we successfully identified epidermal cells, mesophyll cells, bundle sheath cells, epidermis/Fra cells, xylem cells, and phloem cells [32]. However, specific conical cells and parenchyma cells were not identified due to the absence or low expression of the corresponding marker gene expression. The epidermis serves as a site for pigment production. The lower epidermal cells were observed to be lenticular and flatter than the conical cells found on the petals, and they typically contain pigments and may also serve as sites for scent production [39, 40, 42, 43].

We employed *in situ* hybridization experiments to detect the distribution of *RcFRAA3* and *RcHEL*, which have been classified as epidermis/Fra cell markers in rose petal cells. Our findings indicated that these markers were predominantly expressed in the epidermis and vascular tissues, which agrees with previous findings [40, 43]. Furthermore, *RcCML23* was primarily localized in the vascular tissue, corroborating the results of our subgroup classification.

### Effects of *B. cinerea* infection on the epidermal cells of rose petals

The epidermis is the natural physical barrier of the petal to respond to external stress. We observed that, prior to infection, the petal epidermis was in a naturally relaxed state. During the early stage of infection, the epidermal cells exhibited significant swelling, indicating that a substantial amount of water and nutrients had accumulated to counteract external stress. In the intermediate stage of infection, the upper epidermis visibly shrank and ruptured, which may represent a feature characteristic of successful infection by *B. cinerea* into the interior of the petals. The molecular mechanisms underlying the response of rose epidermal cells in this defense process require further investigation.

Among the up-regulated genes in the epidermal cells, HSP26-A, ABP19A, SAT, CYP81E8, and DBR were significantly up-regulated at the T1 stage but down-regulated at the T2 stage. In contrast, only ncbi_121050818, ncbi_112179759, FRAA1A.1, ncbi_112192851 (cold and drought-regulated protein CORA), ncbi_112168074 (metallothionein-like protein 1B, MT) and RBG7 exhibited continuous up-regulation throughout the infection process. Previous reports indicate that plant metallothionein (MT) proteins function as ROS scavenging enzymes in rice [44, 45] and cotton [46], and we predict that this is also the case here in rose.

Rice *OsMT1a*, *OsMT2b*, and cotton *GhMT3a* have been found to demonstrate superoxide and hydroxyl radical scavenging activity *in vitro*. The absence of *OsMT2b* expression promotes epidermal cell death and hypersensitive cell death in rice stems, accelerates H_2_O_2_-mediated internode stomatal formation, and alters *OsMT2b* activity, leading to reduced ROS clearance, which may be a general mechanism for regulating rice cell death [45, 47, 48]. In the halophilic plant *Salicornia brachiata*, the CORA-like gene *SbCDR* in transgenic tobacco exhibited improved relative water content, membrane stability index, and osmotic water potential, along with higher expression of ROS scavenging-related genes under stress conditions [49]. Therefore, CORAs and MTs may represent candidate genes for exploring resistance to fungal diseases.

Meanwhile, of the 20 significantly down-regulated genes identified in epidermal cells (Fig. 4C), KEGG analysis revealed that the chlorophyll a-b binding protein genes (CAB-151, LHCB3) were associated with photosynthesis, while gibberellin 2-beta dioxygenase (GA2OX1) was linked to diterpene biosynthesis. Additionally, pectin methylesterases (PMEs) play crucial roles in various physiological processes, including cell isolation, fruit ripening, internode stem growth, root tip and pollen tube elongation, as well as plant defense against pathogens [50]. Pectinesterase/pectinesterase inhibitor 34 (PME34) exhibits cell wall-modifying enzyme activity in *Arabidopsis* in response to heat stress by regulating the flexibility of the cell wall [51]. Proline-rich proteins (PRPs) are cell wall proteins abundant in proline and hydroxyproline, which play a crucial role in plant responses to drought stress and in the cell wall signaling cascade [52, 53]. The accumulation of proline aids cells in maintaining their water content and swelling potential, thereby supporting normal cellular function. Under stress conditions, plants accumulate proline through the P5CS pathway or by degrading PRP proteins [54–57]. The rapid downregulation of the tomato *SlPRP* gene was found to enhance proline concentrations and improve drought resistance [52]. while the apple *MdPRP6* negatively regulates drought stress by modulating proline accumulation [58]. The downregulation of proline-rich cell wall protein 1 (TPRP-F1) and proline-rich protein DC2.15 (EARLI1) may compromise the defensive capacity of rose petal cell walls [59]. Therefore, we speculate that the downregulated expression of PMEs and PRPs may play an important role in the response of roses to *B. cinerea* stress.

It is worth noting that among the 20 significantly downregulated genes, we identified three genes encoding aquaporins (AQPs): aquaporin TIP1–1, aquaporin PIP2–1, and aquaporin PIP2–7. AQPs are the main intrinsic proteins (MIPs) that facilitate water channels and maintain water balance within and outside the cell [60]. In plants infected with pathogens, H_2_O_2_ is produced in apoplasts and transported by PIP to the cytoplasm, where it activates innate immunity to resist pathogen infection [61–63]. In this study, the genes encoding these three aquaporins were significantly down regulated, suggested that during the process of infection, *B. cinerea* might be able to inhibit the expression of AQPs, lead to unbalance of water supply in rose petals and accelerating the infection rate of the fungus, but the mechanism underlying this remains to be studied.

### Secondary metabolites produced in response to *B. cinerea* infection in rose petals

Plant secondary metabolism serves as a vital component of active defense mechanisms against pests and diseases [64], with the production of terpenes, allergens, and flavonoids representing key defensive responses to pathogens. Among these compounds, flavonoids are particularly significant, as they contribute not only to the development of fruit color and flavor but also to plant defense through their antioxidant, antibacterial, and antifungal properties [65, 66].

Many plant antitoxins are flavonoids, a large class of metabolites derived from phenylpropanoids and polyketides and are present in all plants [67–69]. The accumulation of flavonoids in plants infected with pathogens has been shown to contribute to disease resistance. For instance, the increased accumulation of flavonoids enhances the resistance of *B. cinerea* and prolongs the shelf life of tomatoes [70, 71]. As the primary components responsible for flower coloration, anthocyanins are classified as water-soluble flavonoids [72]. In this study, we annotated several genes from the Fra a family that belong to the Bet v1 superfamily and were associated with allergenic reactions [73, 74].

In the study of strawberries, the anthocyanin content and the expression levels of the genes chalcone synthase (*FaCHS*) and phenylalanine ammonia lyase (*FaPAL*) were found to be upregulated following pathogen infection. However, silencing the Fra a gene led to a decrease in anthocyanin levels, accompanied by downregulation of *FaCHS* and *FaPAL*. This indicates that the Fra gene plays a significant role in strawberry pigment biosynthesis and plant defense responses [75]. In this study, during the early stages of *B. cinerea* infection, rose petals exhibited transient red spots. It is speculated that the high expression of the *RcFra* gene during this phase may have promoted the accumulation of anthocyanins as a temporary response to fungal stress. However, the synthesis of anthocyanins was limited and could not adequately cope with the rapid progression of infection. Red spots were observed only transiently in rose petals *in vitro*, in contrast, in normally growing plants, this appearance can persist longer, probably due to a more robust defense system in living plants, although the underlying mechanisms remain unclear.

Plant chitinase plays a crucial role in resisting fungal pathogens by enhancing resistance to necrotic fungal pathogens. The combination of chitinase and 1,3-beta-glucanase can rapidly kill fungi [76, 77]. This study found that multiple chitinase-encoding genes, specifically EP3 were significantly up-regulated in EC and EC/Fra cells (Fig. 6E), it is worth noting that a significant reduction in the lesion area upon the VGIS of *RcEP3* (Fig. 6G). Combined data analysis, the significant up-regulation of *RcEP3* at T1 infection stage strongly indicates that it may promoted the infection of *B. cinerea*. Therefore, future research deserves to pay more in-depth attention to these genes.

### Effects of *B. cinerea* infection on rose petal mesophyll cells

The petal mesophyll consists of vascular bundles surrounded by parenchyma cells. A significant function of the mesophyll is to supply nutrients to the petal, while the vascular bundles embedded within the parenchyma deliver essential water and metabolites necessary for the petal's function. Additionally, petal mesophyll cells are typically the first site of petal senescence [78–80].

Gas exchange occurs through gaps between the stomata on the petal epidermis and the mesophyll, facilitating photosynthesis for the formation of anthocyanins [81, 82]. In this study, we conducted *in situ* hybridization experiments and found that *RcHEL* and *RcFRAA3* were significantly expressed in the petal epidermis, mesophyll, and vascular tissues (Fig. 7). We speculate that *B. cinerea* primarily enters the petals through the epidermis, from where it diffuses from the vascular tissues to the mesophyll, subsequently blocking the water and nutrient supplies to the rose petals, and leading to petal aging and death.

### Expression of plant disease-related genes in response to *B. cinerea* infection in rose petals

In this study, genes involved in plant–pathogen interaction pathways were significantly expressed in epidermal cells (Figs 3C and 6D). These genes are mainly related to the biosynthesis of cuticular wax, such as CUT1, KCS1, and KCS20. Very-long-chain fatty acids (VLCFAs) are membrane components and act as surface barriers, the first key step in VLCFAs biosynthesis is the condensation of C(2) units into acyl-CoA through 3-ketoacyl-CoA synthase (KCS). In *Arabidopsis*, the complete absence of *KCS20* and *KCS2/DAISY* leads to a decrease in total wax content in stems and leaves, while overexpression increases the total wax content in transgenic leaves [83]. *Arabidopsis KCS9* is involved in the extension of C22 to C24 fatty acids, which is a necessary precursor for the biosynthesis of cuticular waxes, aliphatic suberins, and membrane lipids. However, *Arabidopsis* KCS3 acts as a negative regulator of wax metabolism and can reduce the enzyme activity of KCS6, a key enzyme in wax synthesis, thereby affecting wax synthesis [84, 85]. *Arabidopsis* KCS19-mediated VLCFA synthesis is essential for cuticular wax biosynthesis and seed storage lipids, and it influences the plant's response to abiotic stress [86]. In our study, the significant upregulation of KCSs genes indicates that they highly related to wax synthesis of rose petal and actively respond to *B. cinerea* stress, but the regulatory relationships among them still need further exploration.

In bundle sheath cells, the MAPK signaling pathway was significantly enriched, indicating that pathogen defense-related genes may be mainly differential expressed in it. These genes are related to hormone signaling pathways such as ethylene synthesis and abscisic acid signal, significantly upregulated genes include *RcEBF2*, *RcXRN4*, *RcEIL3, RcPP2CA*, *RcSAPK2*, *RcMPK7*, and *RcMAPKKK18*. Meanwhile, we also identified significantly upregulated genes related to camalexin synthesis and defense response to pathogens, such as *RcWRKY22*, *RcWRKY24*, *RcVIP1*, *RcMKS1*, and *RcACS1*. *AtWRKY29* is a key transcriptional activator involved in the expression of defense genes in the innate immune response of *Arabidopsis thaliana*. *WRKY29* and *WRKY22* may be functionally redundant, and both play roles in defense against fungal and bacterial pathogens [87]. *AtWRKY33* is crucial for resisting the fungus *B. cinerea*, and direct targets in redox homeostasis, salicylic acid signaling, ethylene-jasmonic acid-mediated cross-talk, and glucosinolate biosynthesis [88]. In our study, *RcWRKY33* was mainly expressed in epidermal/fra cells and petal mesophyll cells, but it shows differential expression among the three infection stages of epidermal cells and epidermal/fra cells, which was up-regulated at stage T1, but significantly down-regulated at stage T2. Additionally, as a transcriptional repressor in jasmonic acid signaling, *RcTIFY3B* was significantly expressed in bundle sheath cells, suggesting that it may positively involved in the defense response of rose petals.

There are complex regulatory networks in rose petals during gray mold infection. In tomato, knocking out *SlNPR1* reduced the development of gray mold disease in tomato fruits, and enhanced the activities of glutathione S-transferase (GST), chitinase CHI, and other defense enzymes, which improved the ability of tomato fruits to resist gray mold disease [89]. Methyl jasmonate (MeJA) treatment enhanced the accumulation of total phenols and flavonoids, induced the expression of pathogenic genes (PR), β-1,3-glucanase and chitinase activities, and improved the resistance to *B. cinerea*. However, its role is closely related to the expression of *SlMYC2* [ 90]. These results were consistent with the significant expression of genes encoding GST, CHI, PR, β-1,3-glucanase in our study. Meanwhile, genes such as *RcFras*, *RcGT3*, and *RcGST* may play an important role in the defense response by participating flavonoid synthesis, but the internal regulatory relationship in the process still needs to be further clarified.

### The proposed regulation network of rose petals under *B. cinerea* infection

**Figure 8:**
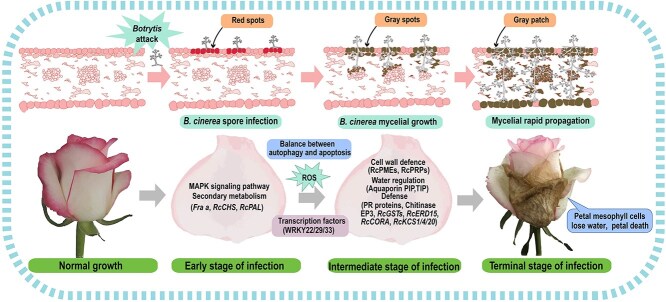
Proposed regulatory network of the *B. cinerea* infection mechanism in rose petals. The rose epidermis is the physical barrier of defense in the early stage of infection. The upregulation of pathogenesis-related protein FRAs promotes the synthesis of anthocyanins, which temporarily alleviates the infection process of the pathogen. When the pathogen penetrates the epidermis, this may accelerate the rate of infection through vascular tissue. At the same time, the fungus inhibits the expression of aquaporin TIP1–1, aquaporin PIP2–1 and aquaporin PIP2–7, thereby blocking the transport of water and nutrients through the petal tissue, and leading to rapid senescence of petal mesophyll and ultimately petal death. Meanwhile, *B. cinerea* infection also leads to down-regulated expression of cell wall-related defense genes such as PMEs and PRPs. Glutathione S-transferase plays a crucial role in cellular detoxification and oxidative stress tolerance in the infection process. Endochitinase EP3 may be promoting in inhibiting fungal spore germination and hyphal growth.

*B. cinerea* is a significant pathogen affecting cut rose production, often resulting in substantial losses and presenting a persistent challenge in the field. Our study identified seven distinct cell groups within rose petals through scRNA-seq and constructed an initial gene regulatory network for *R. hybrida* “Jumilia” petals in response to *B. cinerea* infection (Fig. 8). The rose epidermis serves as the natural physical barrier of defense against *B. cinerea*. During the early stages of infection, the upregulation of pathogenesis-related protein major strawberry allergen FRAs enhances the synthesis of anthocyanins, which temporarily mitigates the infection process. However, once the pathogen penetrates the epidermis, the infection rate may accelerate via vascular tissues and inhibit the expression of aquaporin TIP1-1, aquaporin PIP2-1, and aquaporin PIP2-7. This inhibition disrupts the transport of water and nutrients within the petal tissue, leading to rapid senescence of the petal mesophyll and ultimately resulting in petal death. Meanwhile, *B. cinerea* infection results in the down-regulation of cell wall-related defense genes, such as PMEs and PRPs. Glutathione S-transferase plays a crucial role in cellular detoxification and oxidative stress tolerance during the infection process [91]. Endochitinase EP3 may be involved in promoting fungal spore germination and hyphal growth. However, the underlying mechanisms require further exploration.

## Conclusions

This study explored the impact of *B. cinerea* infection on the petals of the highly susceptible *R. hybrida* “Jumilia” from the single cell level, seven cell subpopulations were identified, and found that the epidermis and vascular tissue are the main parts of the infection. Some key regulatory genes were identified, and through pseudotime analysis, it was discovered that epidermis/Fra-related cells participated in the synthesis of secondary metabolites and play a role in responding to stress, and a gene regulatory network for rose petals in response to *B. cinerea* infection was preliminarily constructed. This preliminary study revealed the cellular changes and molecular mechanisms involved in the process. Our findings provide new insights into the defense mechanisms of roses in response to fungal diseases.

## Materials and methods

### Plants and fungal growth conditions

The experiment focused on the common cut rose variety “Jumilia” cultivated in Yunnan, China. Fresh rose petals were collected from plants that had survived for over six months in the greenhouse of the College of Horticulture and Landscape, Yunnan Agricultural University (Kunming, Yunnan, China). The experimental plants were cultivated in three-liter pots filled with a soilless culture substrate, with one plant per pot. The soilless culture medium consisted of a mixture of peat, coconut coir, and perlite in a ratio of 4:4:1.

The fungus utilized in the experiment was derived from the *B. cinerea* strain KMSC-011, which is preserved by the College of Horticulture and Landscape at Yunnan Agricultural University. The purified gray mold was inoculated onto a petri dish containing PDA medium and subsequently cultured for ten days at a temperature of 25°C and a relative humidity (RH) of 75%.

### Preparation of rose petal samples

A culture dish containing fungal spores was supplemented with 5 ml of sterile water, and spores were scraped from the surface and suspended in the water using sterile bamboo sticks. Impurities were filtered out using a double-layer gauze. The concentration of the spore suspension was determined using a blood cell counting plate and adjusted to 1 × 10^6^ conidia/ml with sterile water. Subsequently, 10 ml of the spore suspension was applied to each petal on a cut rose. The cut roses were then placed in a bottle maintained at 25°C and 90% RH. Following treatment with *B. cinerea*, rose petals display significant disease symptoms, including the appearance of red spots at 36 hours and subsequently black spots at 72 hours. Consequently, tissue samples for RNA sequencing were collected from the rose petals at three time points: before inoculation (0 hours), post-inoculation at 36 hours (red spots), and 72 hours (brown spots). The tissue surrounding decaying lesions, approximately 3 mm wide, along with the pigmented lesion tissue, was excised for experimental analysis. Additionally, equivalent but healthy tissue from clean petals without inoculation was also cut to serve as a control. The growth status of each plant at each sampling point was documented through photography.

### Measurement of physiological indicators

Several physiological traits were measured following the infection of rose petals with *B. cinerea*. The activities of antioxidant enzymes, including superoxide dismutase (SOD), peroxidase (POD), and catalase (CAT), were evaluated using assay kits from Grace Biotechnology (Suzhou, China), in accordance with the manufacturer's instructions [93]. The malondialdehyde (MDA) content was also quantified using an MDA assay kit from the same manufacturer, following the provided protocol [92]. Each trait was assessed in six replicates, and the results were analyzed statistically using Fisher’s least significant difference (LSD) tests. Determination of anthocyanin content as was follows: 100 mg of petal tissue surrounding a lesion was collected and incubated in the dark at −80°C for 10–15 min. The tissue was then ground quickly in liquid nitrogen and 10 ml of a methanol solution containing 1% HCl was added. The mixture was shaken vigorously and allowed to stand at 4°C in the dark for 2 hour, then centrifuged at 10 000 rpm for 10 min. Finally, the supernatant was collected and the absorbance of the extract at 530 and 657 nm measured using a UV spectrophotometer. There were three biological replicates in each group. The delphinidin content was analyzed by the Metware Biotechnology Co., Ltd. (Wuhan, China).

### Preparation of protoplasts

Rose petals at three stages of infection (non-infection as mock(T0), early stage of infection (T1), and intermediate stage of infection (T2)) were rapidly cut into small pieces, the tissue surrounding pigmented lesion tissue, along with the decaying lesions, approximately 5 mm wide, was excised for experimental analysis. Each sample contained 20 pieces of rose petal tissue. The prepared samples were then immersed in 5 ml enzyme solution (ES: 1.5% cellulose R10 0.075 g, 0.5% pectinase 0.025 g, 0.8 M mannitol 3.125 ml, 0.2 M KCl 500 μl, 0.1 M MES (pH 5.7) 500 μl, ddH_2_O 500 μl, 1 M CaCl_2_ 50 ml, 500 × BSA 25 μl) in a vacuum pump at room temperature for 10 min. Then the mixture was placed in vortex mixer for 2–3 hours at 30°C and 75 rpm in the dark, and low-speed oscillated every 30 min to ensure the cell walls were completely hydrolyzed and to fully release the cell contents. The released protoplasts were filtered through a 40-μm cell strainer, and rinsed with 2 ml WB (0.4 M mannitol, 0.5% BSA). The filtrate was centrifuged at 150 g at 4°C for 5 min. The supernatant was discarded, and the precipitate was resuspended by the addition of 2 ml WB and then centrifuged at 150 g at 4°C for 3 min twice. This procedure removed the oversized fragment of tissues and impurities and a pure protoplast suspension was obtained. The protoplast viability was determined with 5 μl 0.4% trypan blue staining. The ratio of viable cells to total cells for samples was >85%, to meet the sequencing requirements. The concentration of protoplasts in each sample was analyzed using a Countess® II Automated Cell Counter and adjusted to 1000~2000 cells/ml. Cells were then placed on the ice for scRNA-seq library construction and sequencing.

### scRNA-seq library construction and sequencing

Single-cell suspensions of cut rose petals were loaded onto a 10× Genomics GemCode Single-cell instrument to generate single-cell gel beads in emulsion (GEMs). The scRNA-seq libraries were prepared using Chromium Single Cell 3′ Reagent Kits (v2), following the manufacturer's instructions. Library sequencing was conducted using a Illumina HiSeq 4000 in a custom paired-end sequencing mode of 26 bp (read 1) × 98 bp (read 2).

### scRNA-seq bioinformatics analysis

The 10× Genomics analysis software Cell Ranger was used to examine the quality of the raw data. Spliced Transcripts Alignment to a Reference alignment software was employed to align Read2 to the *R. chinensis* genome (GCF_002994745.2). Only confidently mapped, non-PCR duplicates with valid barcodes and UMIs were used to generate the gene-barcode matrix. The unique molecular identifiers (UMIs) associated with each gene ID in every barcode were identified and the number of distinct UMIs was calculated to assess the cellular gene expression levels. Following this, ineffective cells were eliminated, and additional quality control and data analysis was conducted using the R package Seurat [93].

Next, *t*-distributed stochastic neighbor embedding (*t*-SNE) was used to project high-dimensional cell data into a 2D space, and SingleR was employed to identify cell types by comparing the similarity of expression patterns between the target cell type and reference cell types. Upregulated genes in each cell subgroup were selected based on a gene expression fold change of log_2_FC ≥ 0.36, *P*-value ≤0.01, and those genes expressed in more than 25% of cells in the target subpopulation. The identification of marker genes was informed using previously reported marker genes and the online resource Plant Cell Marker (https://www.tobaccodb.org/pcmdb/homePage).

### Bulk RNA-seq analysis and correlation analysis

Rose petals at three stages of infection (T0, T1, and T2) were collected. The samples were frozen in liquid nitrogen and stored at −80°C for future use. Total RNA was extracted using the Trizol reagent kit (Invitrogen, Carlsbad, CA, USA) according to the manufacturer's instructions. The enriched mRNA was then fragmented into short segments using fragmentation buffer and subsequently reverse transcribed into cDNA. The resulting cDNA library was sequenced using the Illumina NovaSeq6000 platform by the Gene Denovo Biotechnology Co. (Guangzhou, China). High-quality reads obtained from the sequencing machines were then subjected to bioinformatics analysis. All clean reads were then used in in assembly and gene abundance calculations. Paired-end clean reads were mapped to the *R. chinensis* genome (GCF_002994745.2) using HISAT2. 2.4 [34]. The fragments per kilobase of transcript per million mapped reads (FPKM) was employed to calculate gene expression levels. The correlation coefficient between bulk RNA sequencing (bulkRNAseq) and scRNAseq was determined using Spearman correlation analysis.

### Paraffin section and scanning electron microscopic observation

Fresh petals were collected and immediately fixed using FAA fixation solution, followed by the preparation of paraffin slices. After dewaxing, the samples were immersed in absolute ethanol for 5 min twice, then in 75% alcohol for 5 min, and subsequently washed with running water. The specimens were then placed in a plant safranine dyeing solution for 2 hour, after which they were washed to remove excess dye. They underwent decolorization in 50%, 70%, and 80% alcohol sequentially, before being transferred to a solid green dyeing solution. The samples were dehydrated with absolute ethanol, cleared with xylene, sealed with neutral gum, and examined under a microscope.

The samples were collected and fixed for 2 hour using a specialized electron microscope fixation solution at room temperature. The fixed samples, measuring approximately 3–5 mm, were then stored at 4°C in a dark environment. Each sample was rinsed three times with 0.1 M phosphate buffer (pH 7.4) for 15 min per rinse. The electron microscope fixation solution was applied in the dark for 1–2 hours at room temperature. Following this, the samples were rinsed three times with 0.1 M phosphoric acid buffer (pH 7.4), with each rinse lasting 15 min. Dehydration was achieved using alcohol and isoamyl acetate at varying concentrations for 15 min each. Prepared samples were examined under a scanning electron microscope.

### *In situ* hybridization assay

The samples were collected, stored in liquid nitrogen, and subsequently kept at −80°C for later use. Following tissue removal and washing, the samples were placed in a fixation solution (DEPC water preparation) and fixed for a duration of 2 to 12 hours. The process included immersion, embedding, slicing, roasting, dewaxing, and digestion in a gradient solution after fixation. The sequences of the RNA probes for CML23, FRAA3, and HEL are given in Supplemental Table S8. Using 6-FAM green fluorescence, each gene was hybridized monochromatically. A prehybridization solution was added dropwise and incubated at 37°C for 1 hour. The hybridization solution containing the probe was then added, and the samples were incubated at 37°C for overnight hybridization. After washing, DAPI (4′, 6-diamidino-2-phenylindole) was added and the samples were incubated in the dark for 8 min. An anti-fluorescence quenching tablet was then added to seal the samples, and images were observed under a fluorescence microscope.

### RT-qPCR analysis

Total RNA was extracted from the samples and reverse transcribed into cDNA using a Takara reverse transcriptase M-MLV system. Using a KAPA SYBR Rapid Quantitative PCR Kit (KAPA Biosystems) and following the manufacturer’s instructions, RT-qPCR was performed on a StepOnePlus RealTime PCR system (Thermo Fisher Scientific). *RhACT5* was used as an internal reference gene [4]. The 2^-ΔΔCt^ method was performed for analysis. All primers used for qRT-PCR were listed in Supplemental Table S8.

### Pseudotime analysis

Monocle was used to construct cell differentiation trajectories, allowing for the visualization of the differentiation status of epidermis/Fra cells across three samples through cell trajectory analysis. DEGs were identified based on their expression levels in each differentiation state among the cells (FDR < 1e^−5^), and their distributions were visualized using scatter plots. Additionally, branch-specific DEGs were analyzed (FDR < 1e^−7^), followed by hierarchical clustering of these branch-dependent genes to illustrate trends in gene expression relative to branch expression. Based on gene significance, the top 10 branch-dependent genes were selected to create a trajectory map of differential gene expression over pseudotime.

### Virus-induced gene silencing in rose petals

To construct a silencing vector, specific primers were used to clone the target sequence. Following Cao's method [4], rose petals were punched into circles with a diameter of 1.5 cm using a puncher. Then *Agrobacterium* cultures containing constructs expressing TRV1 and TRV2 were mixed in a 1:1 ratio and vacuum infiltrated into petal discs. The petals were soaked in bacterial solution at 0.05 Mpa for 5 min and then cultured for 2 d. 2 d later, the petals were inoculated with *B. cinerea* for experimentation. The lesion area was measured using ImageJ. All primers used for VIGS were listed in Supplemental Table S8.

## Supplementary Material

Web_Material_uhaf152

## Data Availability

The raw data has been submitted to NCBI SRA database under the project number: SRR32083325–29.

## References

[1] Qi W, Chen X, Fang P. et al. Genomic and transcriptomic sequencing of *Rosa hybrida* provides microsatellite markers for breeding, flower trait improvement and taxonomy studies. BMC Plant Biol. 2018;18:119 29907083 10.1186/s12870-018-1322-5 PMC6003205

[2] Hao Y, Cao X, Ma C. et al. Potential applications and antifungal activities of engineered nanomaterials against gray mold disease agent *Botrytis cinerea* on rose petals. Front Plant Sci. 2017;8:1332 28824670 10.3389/fpls.2017.01332 PMC5539092

[3] Andrew M, Barua R, Short SM. et al. Evidence for a common toolbox based on necrotrophy in a fungal lineage spanning necrotrophs, biotrophs, endophytes, host generalists and specialists. PLoS One. 2012;7:e29943 22253834 10.1371/journal.pone.0029943 PMC3256194

[4] Cao X, Yan H, Liu X. et al. A detached petal disc assay and virus-induced gene silencing facilitate the study of *Botrytis cinerea* resistance in rose flowers. Hortic Res. 2019;6:136 31814989 10.1038/s41438-019-0219-2 PMC6885046

[5] Lü P, Zhang C, Liu J. et al. RhHB1 mediates the antagonism of gibberellins to ABA and ethylene during rose (*Rosa hybrida*) petal senescence. Plant J. 2014;78:578–90 24589134 10.1111/tpj.12494

[6] Muñoz M, Faust JE, Bridges WC. et al. Relationship of pink pigmentation in rose petals and *Botrytis cinerea*. Plant Health Prog. 2020;21:152–6

[7] Kaur S, Tiwari V, Kumari A. et al. Protective and defensive role of anthocyanins under plant abiotic and biotic stresses: an emerging application in sustainable agriculture. J Biotechnol. 2023;361:12–29 36414125 10.1016/j.jbiotec.2022.11.009

[8] Landi M, Tattini M, Gould KS. Multiple functional roles of anthocyanins in plant-environment interactions. Environ Exp Bot. 2015;119:4–17

[9] Cirillo V, D'Amelia V, Esposito M. et al. Anthocyanins are key regulators of drought stress tolerance in tobacco. Biology. 2021;10:139 33578910 10.3390/biology10020139 PMC7916658

[10] Shi L, Li X, Fu Y. et al. Environmental stimuli and phytohormones in anthocyanin biosynthesis: a comprehensive review. Int J Mol Sci. 2023;24:16415 38003605 10.3390/ijms242216415 PMC10671836

[11] Li Z, Ahammed GJ. Plant stress response and adaptation via anthocyanins: a review. Plant Stress. 2023;10:100230

[12] Kovinich N, Kayanja G, Chanoca A. et al. Not all anthocyanins are born equal: distinct patterns induced by stress in *Arabidopsis*. Planta. 2014;240:931–40 24903357 10.1007/s00425-014-2079-1 PMC4200348

[13] Jones JD, Dangl JL. The plant immune system. Nature. 2006;444:323–9 17108957 10.1038/nature05286

[14] Lai Z, Mengiste T. Genetic and cellular mechanisms regulating plant responses to necrotrophic pathogens. Curr Opin Plant Biol. 2013;16:505–12 23859758 10.1016/j.pbi.2013.06.014

[15] Zhang Z, Song Y, Liu CM. et al. Correction: mutational analysis of the ve1 immune receptor that mediates verticillium resistance in tomato. PLoS One. 2019;14:e0220402 31335905 10.1371/journal.pone.0220402 PMC6650063

[16] Liu X, Wang Z, Tian Y. et al. Characterization of wall-associated kinase/wall-associated kinase-like (WAK/WAKL) family in rose (*Rosa chinensis*) reveals the role of *RcWAK4* in botrytis resistance. BMC Plant Biol. 2021;21:526 34758750 10.1186/s12870-021-03307-9 PMC8582219

[17] Wang Z, Ma Y, Chen M. et al. Comparative genomics analysis of WAK/WAKL family in Rosaceae identify candidate WAKs involved in the resistance to *Botrytis cinerea*. BMC Genomics. 2023;24:337 37337162 10.1186/s12864-023-09371-9 PMC10278292

[18] Brutus A, Sicilia F, Macone A. et al. A domain swap approach reveals a role of the plant wall-associated kinase 1 (WAK1) as a receptor of oligogalacturonides. Proc Natl Acad Sci USA. 2010;107:9452–7 20439716 10.1073/pnas.1000675107 PMC2889104

[19] Liu XT, Li DD, Zhang SY. et al. Genome-wide characterization of the rose (*Rosa chinensis*) WRKY family and role of *RcWRKY41* in gray mold resistance. BMC Plant Biol. 2019;19:522 31775626 10.1186/s12870-019-2139-6 PMC6882016

[20] Ren H, Bai M, Sun J. et al. RcMYB84 and RcMYB123 mediate jasmonate-induced defense responses against *Botrytis cinerea* in rose (*Rosa chinensis*). Plant J. 2020;103:1839–49 32524706 10.1111/tpj.14871

[21] Ullah I, Yuan W, Uzair M. et al. Molecular characterization of bHLH transcription factor family in rose (*Rosa chinensis* Jacq.) under *Botrytis cinerea* infection. Horticulturae. 2022;8:989

[22] Bawa G, Liu Z, Yu X. et al. Single-cell RNA sequencing for plant research: insights and possible benefits. Int J Mol Sci. 2022;23:4497 35562888 10.3390/ijms23094497 PMC9100049

[23] Carter RA, Bihannic L, Rosencrance C. et al. A single-cell transcriptional atlas of the developing murine cerebellum. Curr Biol. 2018;28:2910–2920.e2 30220501 10.1016/j.cub.2018.07.062

[24] Maniatis S, Aijo T, Vickovic S. et al. Spatiotemporal dynamics of molecular pathology in amyotrophic lateral sclerosis. Science. 2018;364:89–93 10.1126/science.aav9776 30948552

[25] Maynard KE, Collado-Torres L, Weber LM. et al. Transcriptome-scale spatial gene expression in the human dorsolateral prefrontal cortex. Nat Neurosci. 2020;24:425–36 10.1038/s41593-020-00787-0 PMC8095368 33558695

[26] Sun X, Feng D, Liu M. et al. Single-cell transcriptome reveals dominant subgenome expression and transcriptional response to heat stress in Chinese cabbage. Genome Biol. 2022;23:1–19 36536447 10.1186/s13059-022-02834-4 PMC9762029

[27] Sun S, Shen X, Li Y. et al. Single-cell RNA sequencing provides a high-resolution roadmap for understanding the multicellular compartmentation of specialized metabolism. Nat Plants. 2023;9:179–90 36522449 10.1038/s41477-022-01291-y

[28] Denyer T, Ma X, Klesen S. et al. Spatiotemporal developmental trajectories in the *Arabidopsis* root revealed using high-throughput single-cell RNA sequencing. Dev Cell. 2019;48:840–852.e5 30913408 10.1016/j.devcel.2019.02.022

[29] Kang M, Choi Y, Kim H. et al. Single-cell RNA-sequencing of *Nicotiana attenuata* corolla cells reveals the biosynthetic pathway of a floral scent. New Phytol. 2022;234:527–44 35075650 10.1111/nph.17992 PMC9305527

[30] Bai Y, Liu H, Lyu H. et al. Development of a single-cell atlas for woodland strawberry (*Fragaria vesca*) leaves during early *Botrytis cinerea* infection using single-cell RNA-seq. Hortic Res. 2022;9:uhab055 35043166 10.1093/hr/uhab055 PMC8969069

[31] Guo Y, Chen X, Li J. et al. Single-cell RNA sequencing reveals a high-resolution cell atlas of petals in *Prunus mume* at different flowering development stages. Hortic Res. 2024;11:uhae189 39247887 10.1093/hr/uhae189 PMC11377181

[32] Cavallini-Speisser Q, Morel P, Monniaux M. Petal cellular identities. Front Plant Sci. 2021;12:745507 34777425 10.3389/fpls.2021.745507 PMC8579033

[33] Endress PK, Matthews ML. Elaborate petals and staminodes in eudicots: diversity, function, and evolution. Org Divers Evol. 2006;6:257–93

[34] Moyroud E, Glover BJ. The evolution of diverse floral morphologies. Curr Biol. 2017;27:R941–51 28898667 10.1016/j.cub.2017.06.053

[35] Endress PK . Origins of flower morphology. J Exp Zool. 2001;291:105–15 11479912 10.1002/jez.1063

[36] Veloso J, Van Kan JAL. Many shades of grey in *botrytis*–host plant interactions. Trends Plant Sci. 2018;23:613–22 29724660 10.1016/j.tplants.2018.03.016

[37] Bi K, Liang Y, Mengiste T. et al. Killing softly: a roadmap of *Botrytis cinerea* pathogenicity. Trends Plant Sci. 2023;28:211–22 36184487 10.1016/j.tplants.2022.08.024

[38] Eizner E, Ronen M, Gur Y. et al. Characterization of botrytis-plant interactions using PathTrack© -an automated system for dynamic analysis of disease development. Mol Plant Pathol. 2016;18:503–12 27061637 10.1111/mpp.12410 PMC6638221

[39] Gorton HL, Vogelmann TC. Effects of epidermal cell shape and pigmentation on optical properties of *antirrhinum* petals at visible and ultraviolet wavelengths. Plant Physiol. 1996;112:879–88 12226425 10.1104/pp.112.3.879 PMC158014

[40] Whitney HM, Bennett KM, Dorling M. et al. Why do so many petals have conical epidermal cells? Ann Bot. 2011;108:609–16 21470973 10.1093/aob/mcr065 PMC3170151

[41] Whitney HM, Poetes R, Steiner U. et al. Determining the contribution of epidermal cell shape to petal wettability using isogenic *antirrhinum* lines. PLoS One. 2011;6:e17576 21423738 10.1371/journal.pone.0017576 PMC3053357

[42] Baumann K, Perez-Rodriguez M, Bradley D. et al. Control of cell and petal morphogenesis by R2R3 MYB transcription factors. Development. 2007;134:1691–701 17376813 10.1242/dev.02836

[43] Whitney HM, Chittka L, Bruce TJ. et al. Conical epidermal cells allow bees to grip flowers and increase foraging efficiency. Curr Biol. 2009;19:948–53 19446458 10.1016/j.cub.2009.04.051

[44] Kumar G, Kushwaha HR, Panjabi-Sabharwal V. et al. Clustered metallothionein genes are co-regulated in rice and ectopic expression of OsMT1e-P confers multiple abiotic stress tolerance in tobacco via ROS scavenging. BMC Plant Biol. 2012;12:107 22780875 10.1186/1471-2229-12-107 PMC3491035

[45] Wong HL, Sakamoto T, Kawasaki T. et al. Down-regulation of metallothionein, a reactive oxygen scavenger, by the small GTPase osRac1 in rice. Plant Physiol. 2004;135:1447–56 15220467 10.1104/pp.103.036384 PMC519061

[46] Xue T, Li X, Zhu W. et al. Cotton metallothionein GhMT3a, a reactive oxygen species scavenger, increased tolerance against abiotic stress in transgenic tobacco and yeast. J Exp Bot. 2009;60:339–49 19033550 10.1093/jxb/ern291 PMC3071772

[47] Gao C, Gao K, Yang H. et al. Genome-wide analysis of metallothionein gene family in maize to reveal its role in development and stress resistance to heavy metal. Biol Res. 2022;55:1 35012672 10.1186/s40659-021-00368-w PMC8751047

[48] Steffens B, Sauter M. Epidermal cell death in rice is confined to cells with a distinct molecular identity and is mediated by ethylene and H_2_O_2_ through an autoamplified signal pathway. Plant Cell. 2009;21:184–96 19141708 10.1105/tpc.108.061887 PMC2648082

[49] Jha RK, Patel J, Patel MK. et al. Introgression of a novel cold and drought regulatory-protein encoding CORA-like gene, SbCDR, induced osmotic tolerance in transgenic tobacco. Physiol Plant. 2021;172:1170–88 33206416 10.1111/ppl.13280

[50] Levesque-Tremblay G, Pelloux J, Braybrook SA. et al. Tuning of pectin methylesterification: consequences for cell wall biomechanics and development. Planta. 2015;242:791–811 26168980 10.1007/s00425-015-2358-5

[51] Huang YC, Wu HC, Wang YD. et al. PECTIN METHYLESTERASE34 contributes to heat tolerance through its role in promoting stomatal movement. Plant Physiol. 2017;174:748–63 28381503 10.1104/pp.17.00335 PMC5462046

[52] Gujjar RS, Karkute SG, Rai A. et al. Proline-rich proteins may regulate free cellular proline levels during drought stress in tomato. Curr Sci. 2018;114:915–20

[53] Liu DQ, Han Q, Shah T. et al. A hybrid proline-rich cell-wall protein gene JsPRP1 from *Juglans sigillata* dode confers both biotic and abiotic stresses in transgenic tobacco plants. Trees. 2018;32:1199–209

[54] Pérez-Arellano I, Carmona-álvarez F, Martínez AI. et al. Pyrroline-5-carboxylate synthase and proline biosynthesis: from osmotolerance to rare metabolic disease. Protein Sci. 2010;19:372–82 20091669 10.1002/pro.340 PMC2866264

[55] Barthakur S, Babu V, Bansa KC. Over-expression of osmotin induces proline accumulation and confers tolerance to osmotic stress in transgenic tobacco. J Plant Biochem Biotechnol. 2001;10:31–7

[56] Gujjar RS, Pathak AD, Karkute SG. et al. Multifunctional proline rich proteins and their role in regulating cellular proline content in plants under stress. Biol Plant. 2019;63:448–54

[57] Stines AP, Naylor DJ, Høj PB. et al. Proline accumulation in developing grapevine fruit occurs independently of changes in the levels of Δ1-pyrroline-5-carboxylate synthetase mRNA or protein. Plant Physiol. 1999;120:923–31 10398729 10.1104/pp.120.3.923 PMC59332

[58] Zhang XL, Gong XQ, Su XJ. et al. The ubiquitin-binding protein MdRAD23D1 mediates drought response by regulating degradation of the proline-rich protein MdPRP6 in apple (*Malus domestica*). Plant Biotechnol J. 2023;21:1560–76 37140026 10.1111/pbi.14057 PMC10363924

[59] Li S, Zhang Y, Ding C. et al. Proline-rich protein gene PdPRP regulates secondary wall formation in poplar. J Plant Physiol. 2019;233:58–72 30599461 10.1016/j.jplph.2018.12.007

[60] Tang H, Su Y, Yang S. et al. Aquaporin-mediated stress signaling cascade in plants. Plant Stress. 2023;10:100305

[61] Tian S, Wang X, Li P. et al. Plant aquaporin atPIP1;4 links apoplastic H_2_O_2_ induction to disease immunity pathways. Plant Physiol. 2016;171:1635–50 26945050 10.1104/pp.15.01237 PMC4936539

[62] Zhang M, Shi H, Li N. et al. Aquaporin osPIP2;2 links the H_2_O_2_ signal and a membrane-anchored transcription factor to promote plant defense. Plant Physiol. 2021;188:2325–41 10.1093/plphys/kiab604 PMC8968290 34958388

[63] Rodrigues O, Reshetnyak G, Grondin A. et al. Aquaporins facilitate hydrogen peroxide entry into guard cells to mediate ABA- and pathogen-triggered stomatal closure. Proc Natl Acad Sci USA. 2017;114:201704754 10.1073/pnas.1704754114 PMC5576802 28784763

[64] Badmi R, Gogoi A, Doyle PB. Secondary metabolites and their role in strawberry defense. Plants. 2023;12:3240 37765404 10.3390/plants12183240 PMC10537498

[65] Tariq H, Asif S, Andleeb A. et al. Flavonoid production: current trends in plant metabolic engineering and de novo microbial production. Metabolites. 2023;13:124 36677049 10.3390/metabo13010124 PMC9864322

[66] Dias MC, Pinto DCGA, Silva AMS. Plant flavonoids: chemical characteristics and biological activity. Molecules. 2021;26:5377 34500810 10.3390/molecules26175377 PMC8434187

[67] Tohge T, de Souza LP, Fernie AR. Current understanding of the pathways of flavonoid biosynthesis in model and crop plants. J Exp Bot. 2017;68:4013–28 28922752 10.1093/jxb/erx177

[68] Perez de Souza L, Garbowicz K, Brotman Y. et al. The acetate pathway supports flavonoid and lipid biosynthesis in *Arabidopsis*. Plant Physiol. 2020;182:857–69 31719153 10.1104/pp.19.00683 PMC6997690

[69] Ube N, Katsuyama Y, Kariya K. et al. Identification of methoxylchalcones produced in response to CuCl_2_ treatment and pathogen infection in barley. Phytochemistry. 2021;184:112650 33529859 10.1016/j.phytochem.2020.112650

[70] Zhang Y, De Stefano R, Robine M. et al. Different reactive oxygen species scavenging properties of flavonoids determine their abilities to extend the shelf life of tomato. Plant Physiol. 2015;169:1568–83 26082399 10.1104/pp.15.00346 PMC4634045

[71] Zhang Y, Butelli E, De Stefano R. et al. Anthocyanins double the shelf life of tomatoes by delaying overripening and reducing susceptibility to gray mold. Curr Biol. 2013;23:1094–100 23707429 10.1016/j.cub.2013.04.072 PMC3688073

[72] Sasaki N, Nishizaki Y, Ozeki Y. et al. The role of acyl-glucose in anthocyanin modifications. Molecules. 2014;19:18747–66 25405291 10.3390/molecules191118747 PMC6271837

[73] Ishibashi M, Nabe T, Nitta Y. et al. Analysis of major paralogs encoding the Fra a 1 allergen based on their organ-specificity in *Fragaria×ananassa*. Plant Cell Rep. 2018;37:411–24 29177844 10.1007/s00299-017-2237-6

[74] Karlsson AL, Alm R, Ekstrand B. et al. Bet v 1 homologues in strawberry identified as IgE-binding proteins and presumptive allergens. Allergy. 2004;59:1277–84 15507096 10.1111/j.1398-9995.2004.00585.x

[75] Muñoz C, Hoffmann T, Escobar NM. et al. The strawberry fruit Fra a allergen functions in flavonoid biosynthesis. Mol Plant. 2010;3:113–24 19969523 10.1093/mp/ssp087

[76] Jayaraj J, Punja ZK. Combined expression of chitinase and lipid transfer protein genes in transgenic carrot plants enhances resistance to foliar fungal pathogens. Plant Cell Rep. 2007;26:1539–46 17508215 10.1007/s00299-007-0368-x

[77] Mauch F, Mauch-Mani B, Boller T. Antifungal hydrolases in pea tissue: II. Inhibition of fungal growth by combinations of chitinase and beta-1,3-glucanase. Plant Physiol. 1988;88:936–42 16666407 10.1104/pp.88.3.936 PMC1055685

[78] van Doorn WG, Woltering EJ. Physiology and molecular biology of petal senescence. J Exp Bot. 2008;59:453–80 18310084 10.1093/jxb/erm356

[79] van der Kooi CJ, Stavenga DG. Vividly coloured poppy flowers due to dense pigmentation and strong scattering in thin petals. J Comp Physiol. 2019;205:363–72 30689019 10.1007/s00359-018-01313-1 PMC6579775

[80] Mursidawati S, Wicaksono A, Teixeira da Silva JA. Rafflesia patma Blume flower organs: histology of the epidermis and vascular structures, and a search for stomata. Planta. 2020;251:112 32494866 10.1007/s00425-020-03402-5

[81] Moscovici S, Moalem-Beno D, Weiss D. The role of light reactions in the regulation of anthocyanin synthesis in *petunia corollas*. Physiol Plant. 1991;81:127–33

[82] Weiss D, Shomer-Ilan A, Vainstein A. et al. Photosynthetic carbon fixation in the corollas of *Petunia hybrida*. Physiol Plant. 1990;78:345–50

[83] Lee SB, Jung SJ, Go YS. et al. Two *Arabidopsis* 3-ketoacyl CoA synthase genes, KCS20 and KCS2/DAISY, are functionally redundant in cuticular wax and root suberin biosynthesis, but differentially controlled by osmotic stress. Plant J. 2009;60:462–75 19619160 10.1111/j.1365-313X.2009.03973.x

[84] Huang H, Yang X, Zheng M. et al. An ancestral role for 3-KETOACYL-COA SYNTHASE3 as a negative regulator of plant cuticular wax synthesis. Plant Cell. 2023;35:2251–70 36807983 10.1093/plcell/koad051 PMC10226574

[85] Millar AA, Clemens S, Zachgo S. et al. CUT1, an *Arabidopsis* gene required for cuticular wax biosynthesis and pollen fertility, encodes a very-long-chain fatty acid condensing enzyme. Plant Cell. 1999;11:825–38 10330468 10.1105/tpc.11.5.825 PMC144219

[86] Luo N, Wang Y, Liu Y. et al. 3-ketoacyl-CoA synthase 19 contributes to the biosynthesis of seed lipids and cuticular wax in *Arabidopsis* and abiotic stress tolerance. Plant Cell Environ. 2024;47:4599–614 39041727 10.1111/pce.15054

[87] Asai T, Tena G, Plotnikova J. et al. MAP kinase signalling cascade in *Arabidopsis* innate immunity. Nature. 2002;415:977–83 11875555 10.1038/415977a

[88] Birkenbihl RP, Diezel C, Somssich IE. *Arabidopsis* is a key transcriptional regulator of hormonal and metabolic responses toward *Botrytis cinerea* infection. Plant Physiol. 2012;159:266–85 22392279 10.1104/pp.111.192641 PMC3375964

[89] Li R, Li Y, Zhang Y. et al. Transcriptome analysis reveals that SlNPR1 mediates tomato fruit resistance against *Botrytis cinerea* by modulating phenylpropanoid metabolism and balancing ROS homeostasis. Postharvest Biol Technol. 2021;172:111382

[90] Min D, Li F, Cui X. et al. SlMYC2 are required for methyl jasmonate-induced tomato fruit resistance to *Botrytis cinerea*. Food Chem. 2020;310:125901 31816533 10.1016/j.foodchem.2019.125901

[91] Liu L, Zheng S, Yang D. et al. Genome-wide in silico identification of glutathione S-transferase (GST) gene family members in fig (*Ficus carica* L.) and expression characteristics during fruit color development. PeerJ. 2023;11:e14406 36718451 10.7717/peerj.14406 PMC9884035

[92] Zhou Q, Song S, Wang X. et al. Effects of drought stress on flowering soybean physiology under different soil conditions. Plant Soil Environ. 2022;68:487–98

[93] Butler A, Hoffman P, Smibert P. et al. Integrating single-cell transcriptomic data across different conditions, technologies, and species. Nat Biotechnol. 2018;36:411–20 29608179 10.1038/nbt.4096 PMC6700744

