# Supplementary material for: Single-cell transcriptomic analyses reveal cellular and molecular patterns of rose petal responses to gray mold infection
Source: Hortic Res. 2025 Jun 9;12(9):uhaf152. doi: 10.1093/hr/uhaf152 (PMC12317188; doi:10.1093/hr/uhaf152)
Supplement: Web_Material_uhaf152 [file web_material_uhaf152.zip › Supplemental figure legend.docx]

**
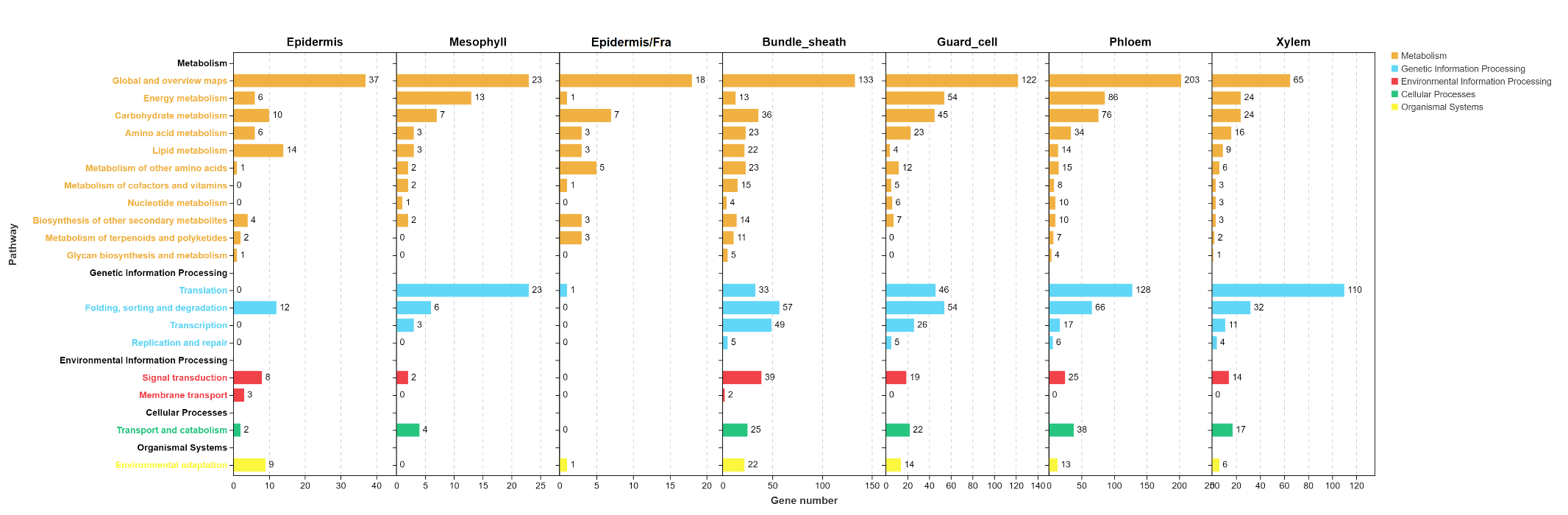
**

**Figure S1. Enrichment analysis of KEGG pathway in 7 subgroup of cells.**

**
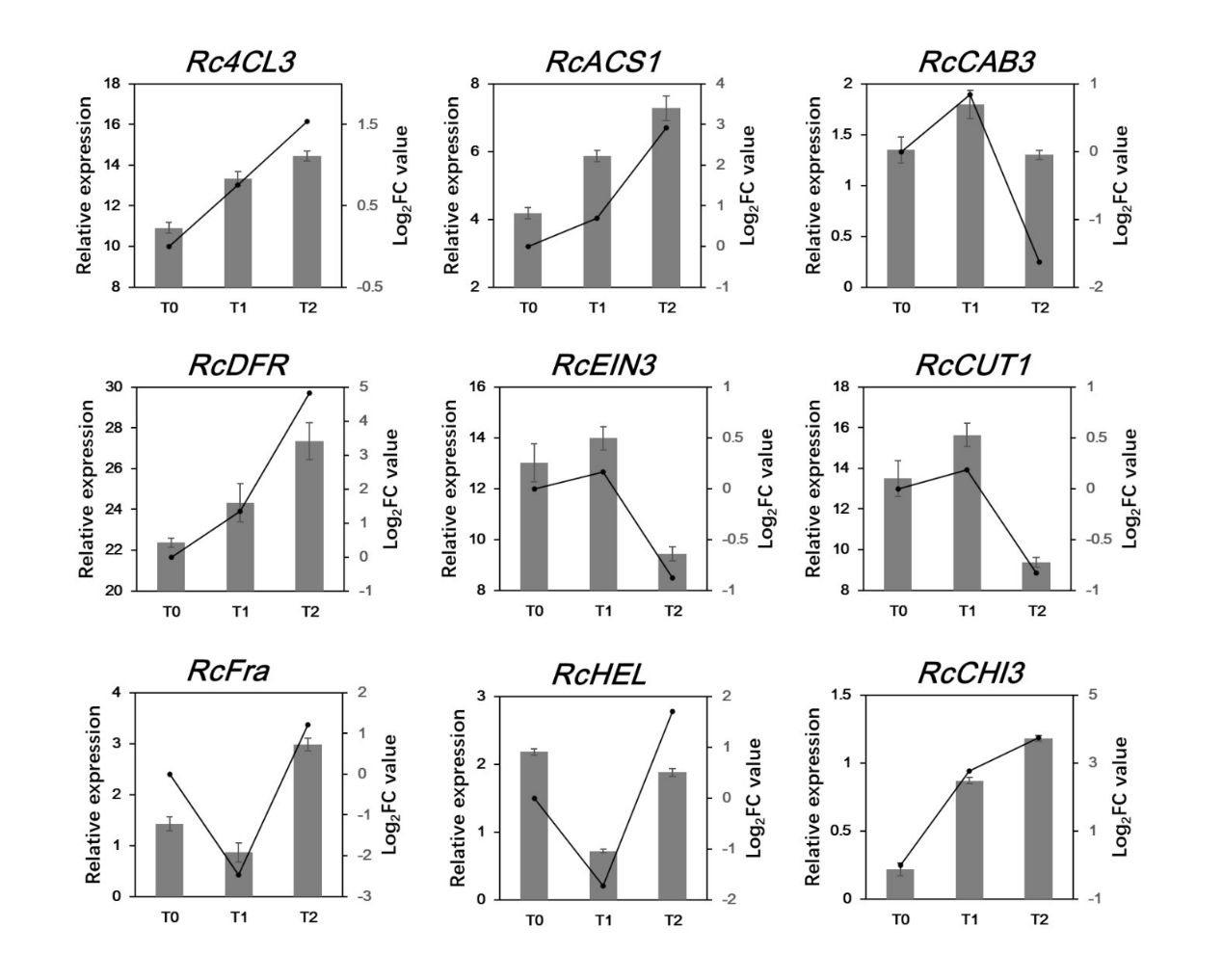
**

**Figure S2. QRT PCR validation of significantly differentially expressed genes corresponding to bulk RNA sequencing**

**
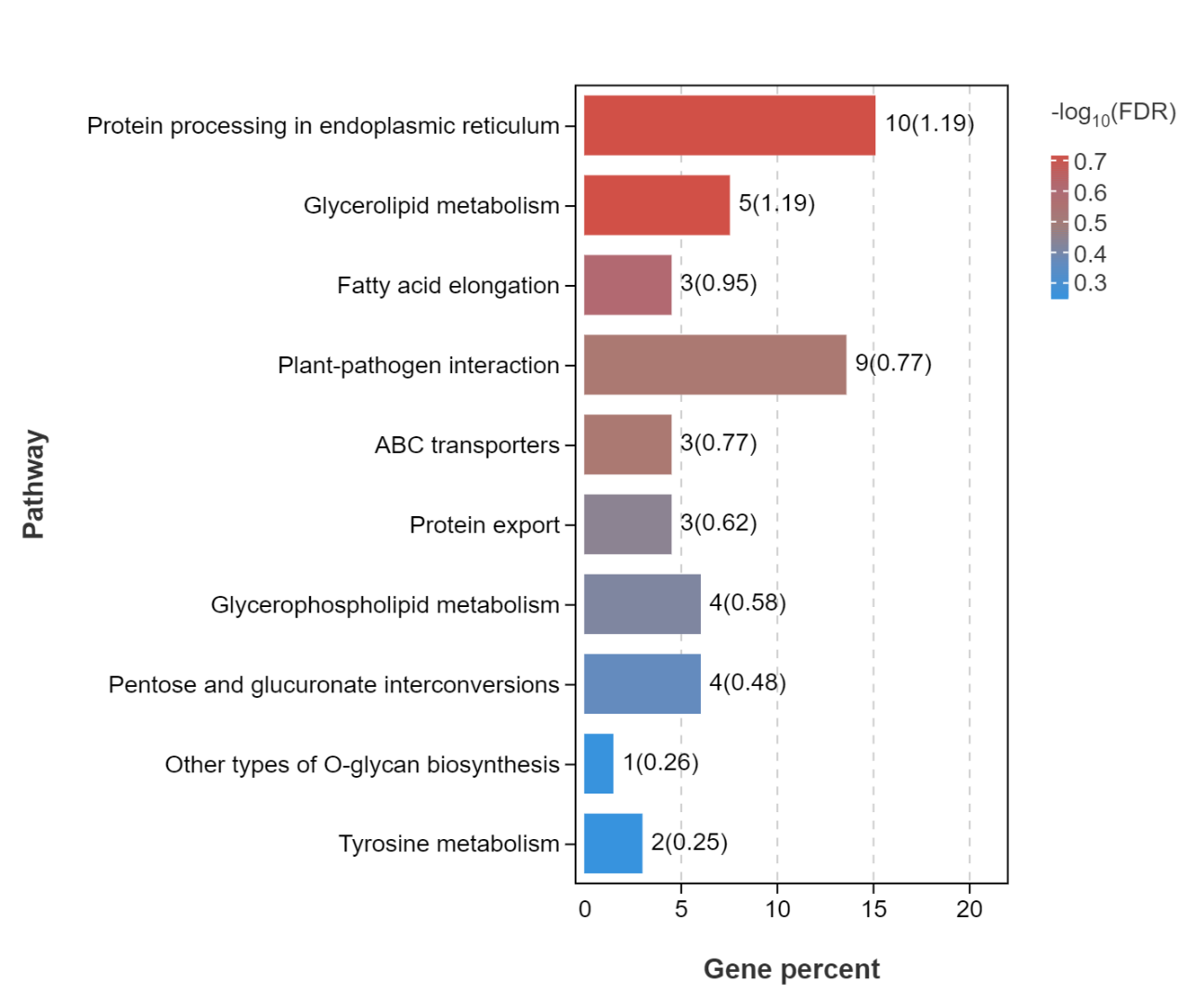
**

**Figure S3. The KEGG pathways analysis of the up-regulated genes in rose epidermal cells**

**
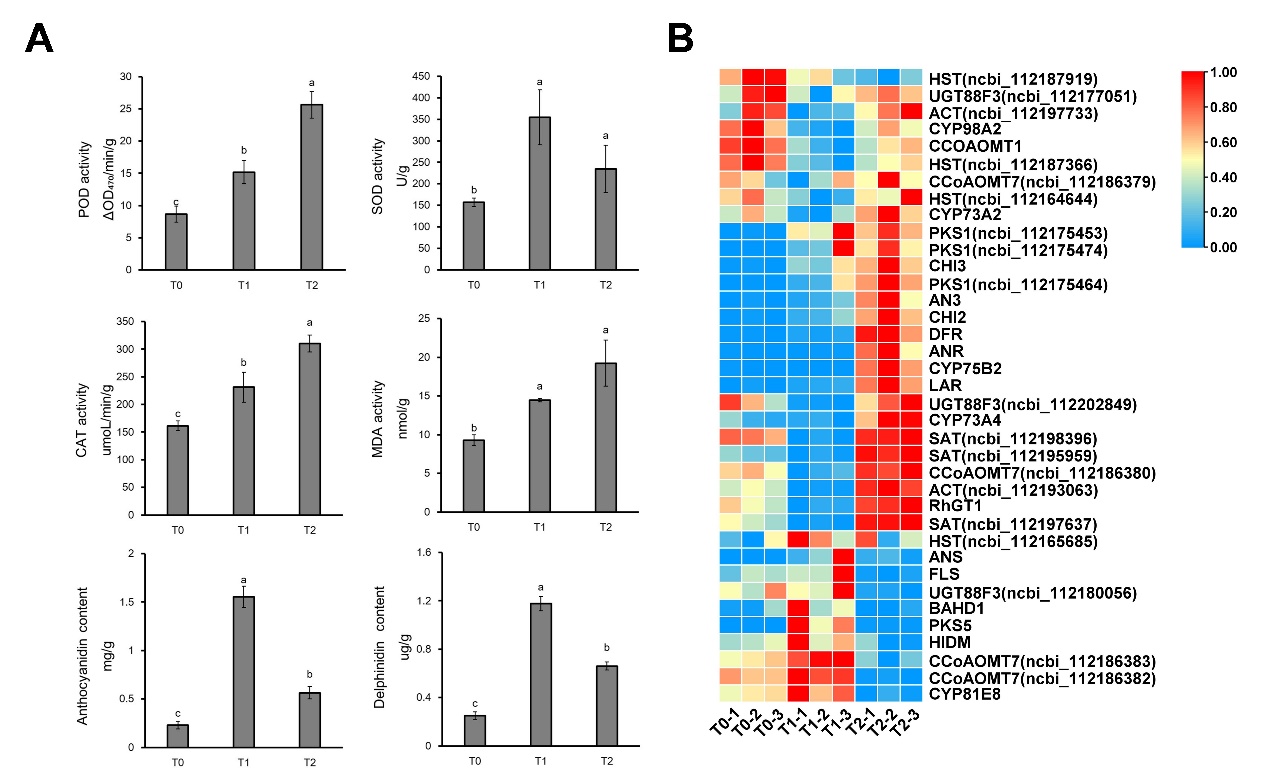
**

**Figure S4.** **Physiological indicators and gene expression analysis of flavonoid biosynthesis in rose petals infected by *Botrytis cinerea.***

**(A)** The changes of physiological indicators and contents of flavonoids-related compounds in three rose petal samples.

**(B**) Heatmap of gene expression related to flavonoid biosynthesis pathway in three rose petal samples.

**
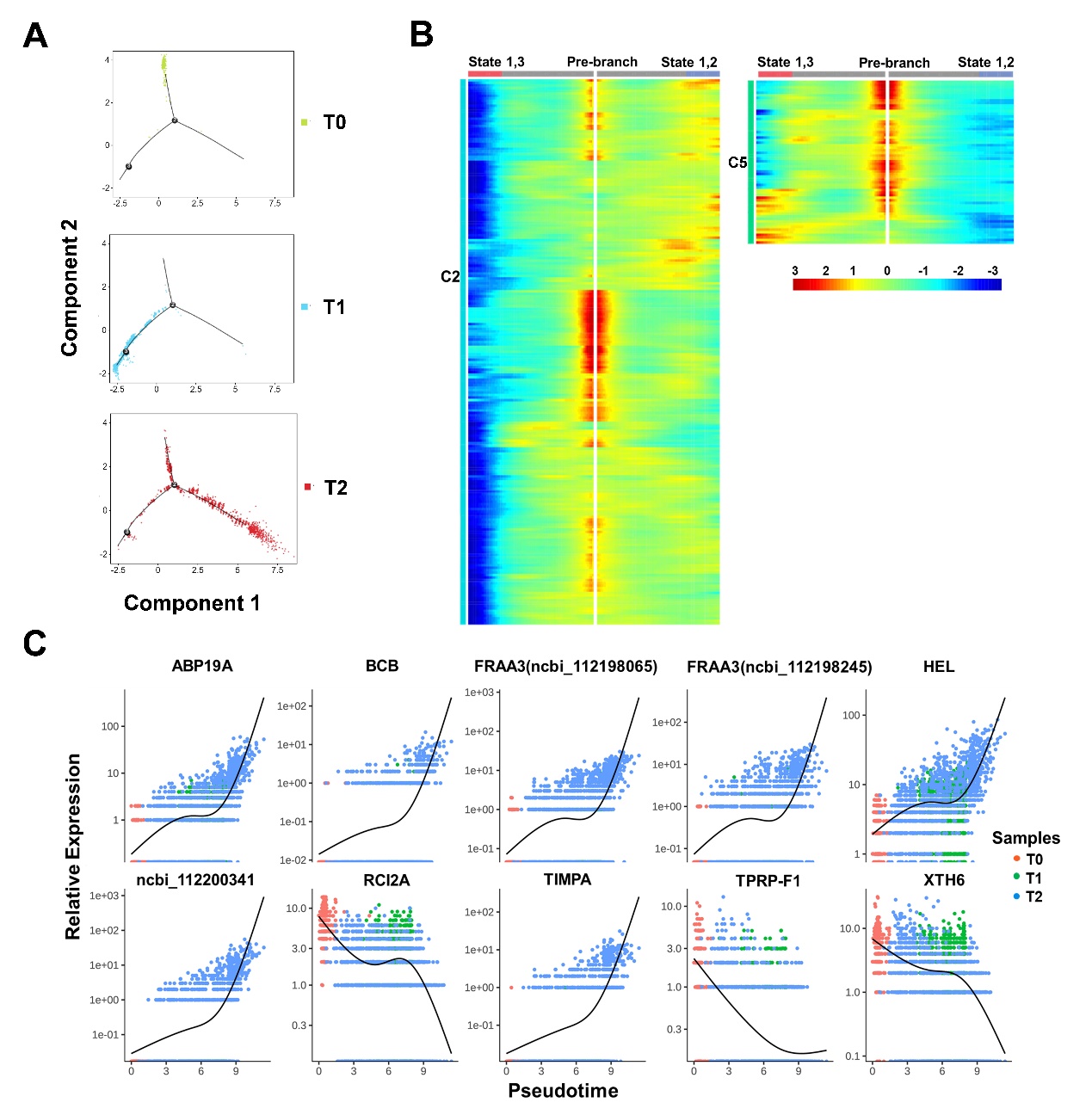
**

**Figure S5. Pseudo-time analysis and expression of DEGs in epidermis/Fra cells among the three stage of rose petals.**

**(A)** Distribution of cells from three stage of samples in Pseudo-time analysis.

**(B)** The heatmap of differentiated fate genes during the process of epidermis/Fra cell differentiation. The cluster heatmap were showed cluster C2 and C5 in the 5 clusters. **(C)** The pseudo-time expression trajectory of significantly differential genes among the three samples. The horizontal axis represents the pseudo-time point, the vertical axis represents the gene expression level, the black solid line represents the fitted line of gene expression level, and different colors represent three samples.

**
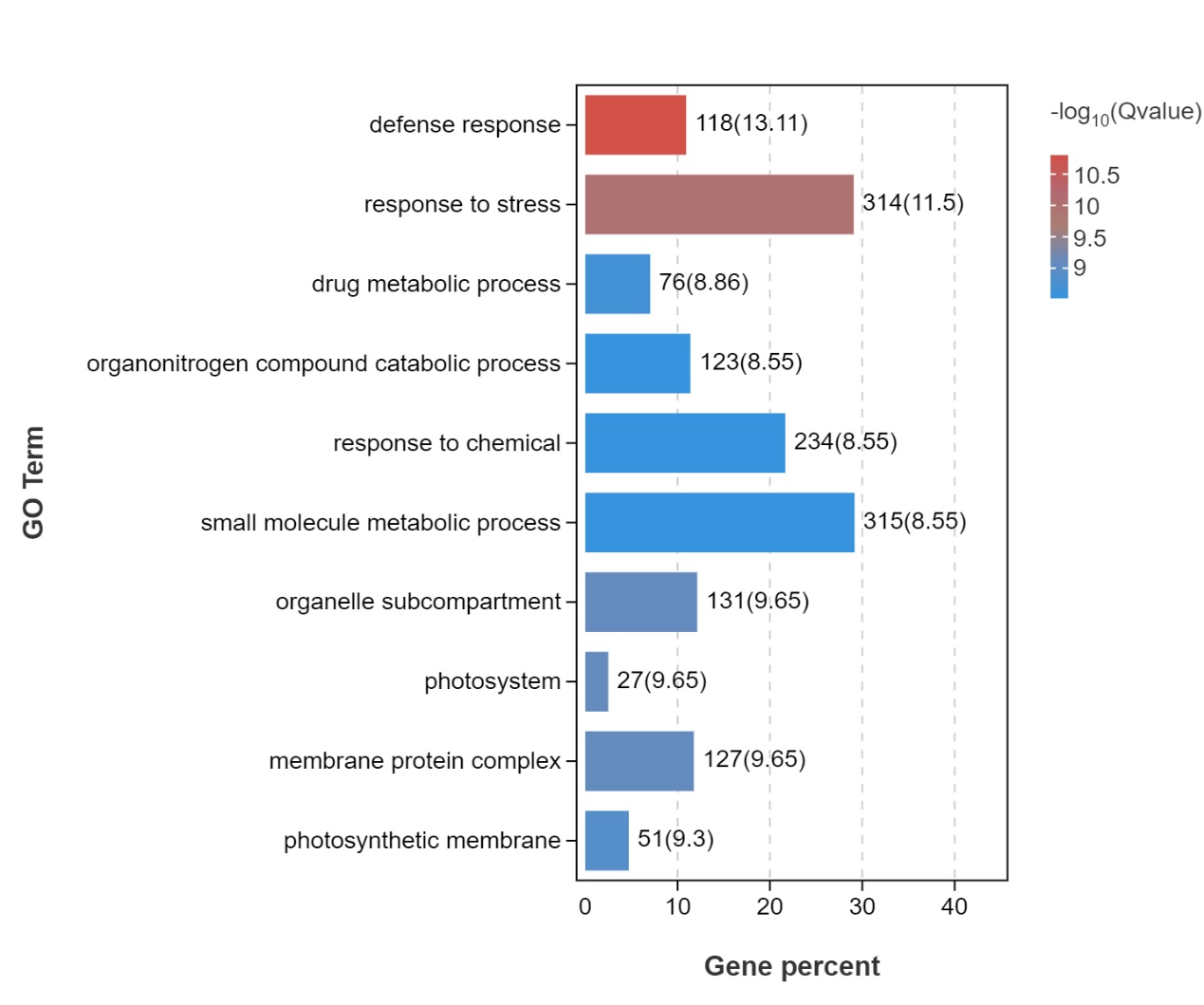
**

**Figure S6. The top10 terms of GO analyses in petal mesophyll cells**

**
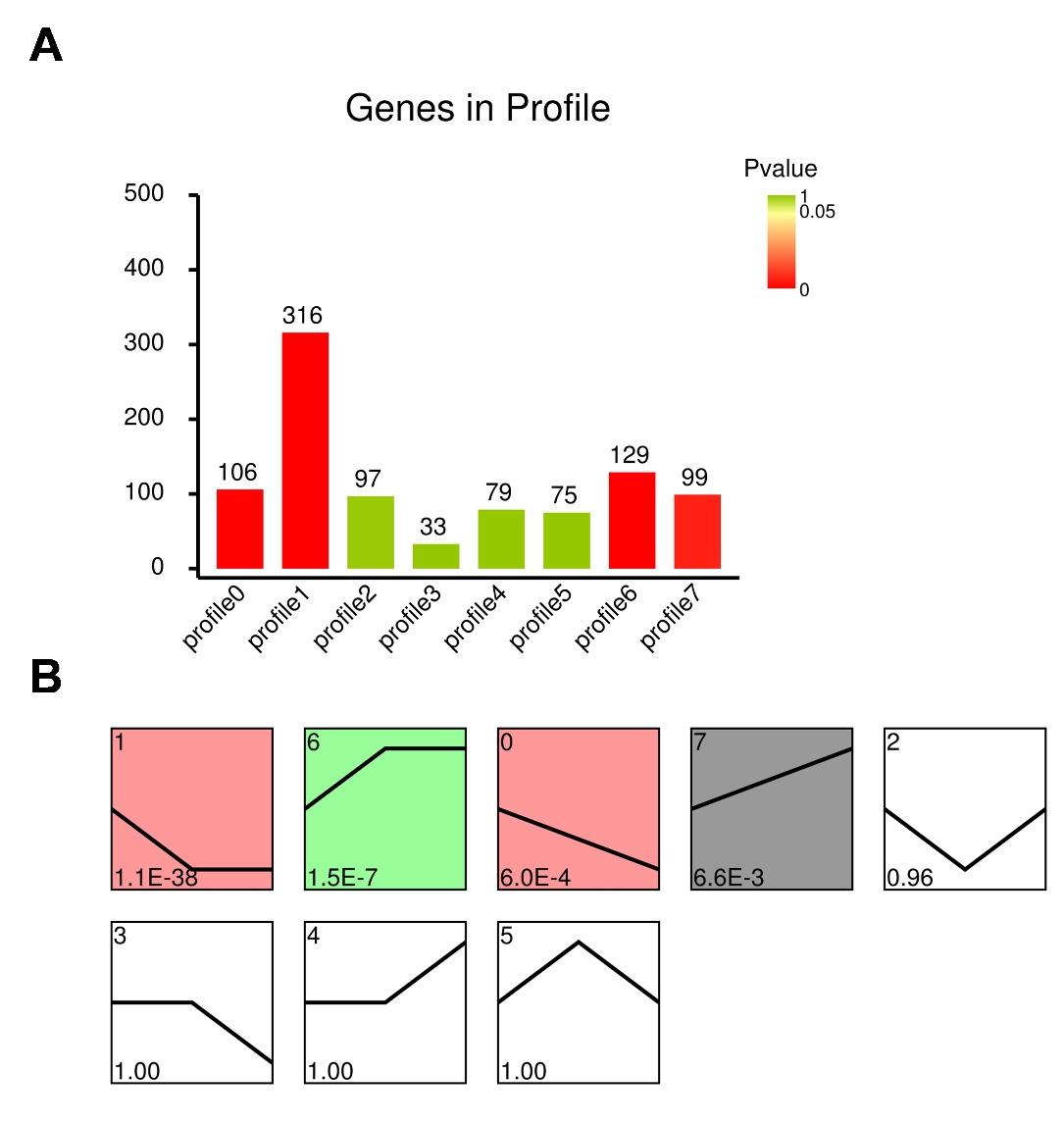
**

**Figure S7. The trend analysis of gene expression in three stages samples under *B. cinerea* infection.**

**(A)** The number of genes in the eight profiles based on the expression trend.

**(B)** The analysis categorizing the expression responses into eight distinct expression trends, the profiles 1, 6, 0 and 7 were defined as significant profiles based on P value < 0.05.
