# Supplementary figures and images for: Single-cell transcriptomic analyses reveal cellular and molecular patterns of rose petal responses to gray mold infection
Source: Hortic Res. 2025 Jun 9;12(9):uhaf152. doi: 10.1093/hr/uhaf152 (PMC12317188; doi:10.1093/hr/uhaf152)

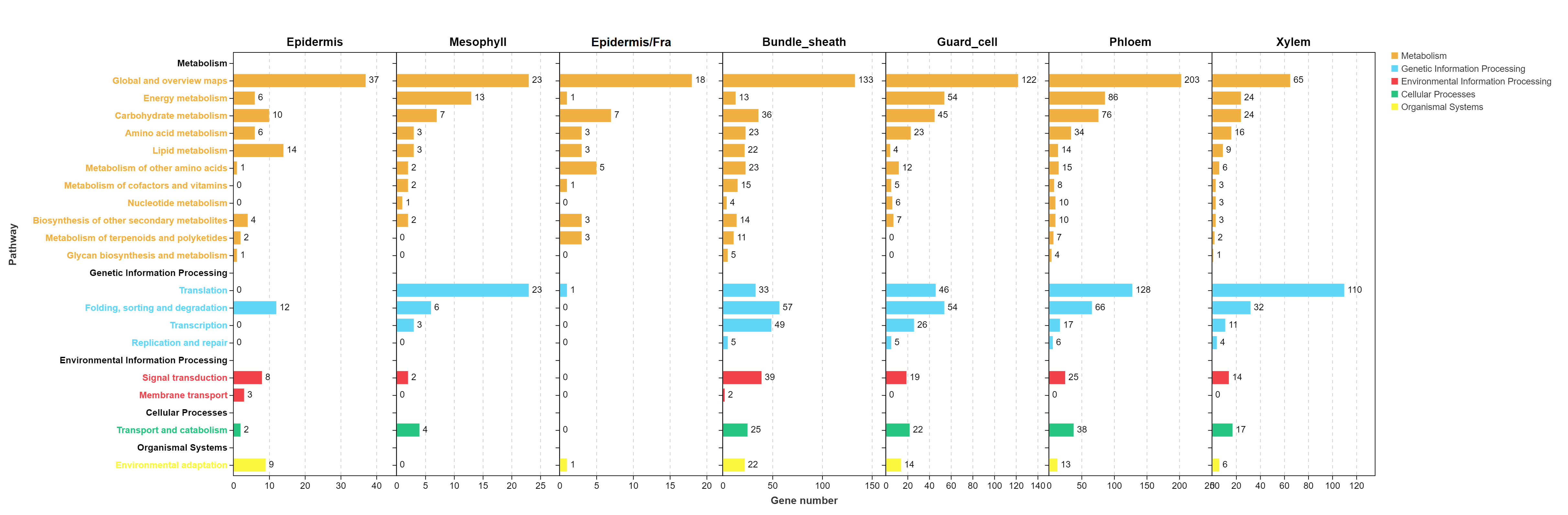

Supplement: Web_Material_uhaf152 [file web_material_uhaf152.zip › Supplementary figure S1.png]

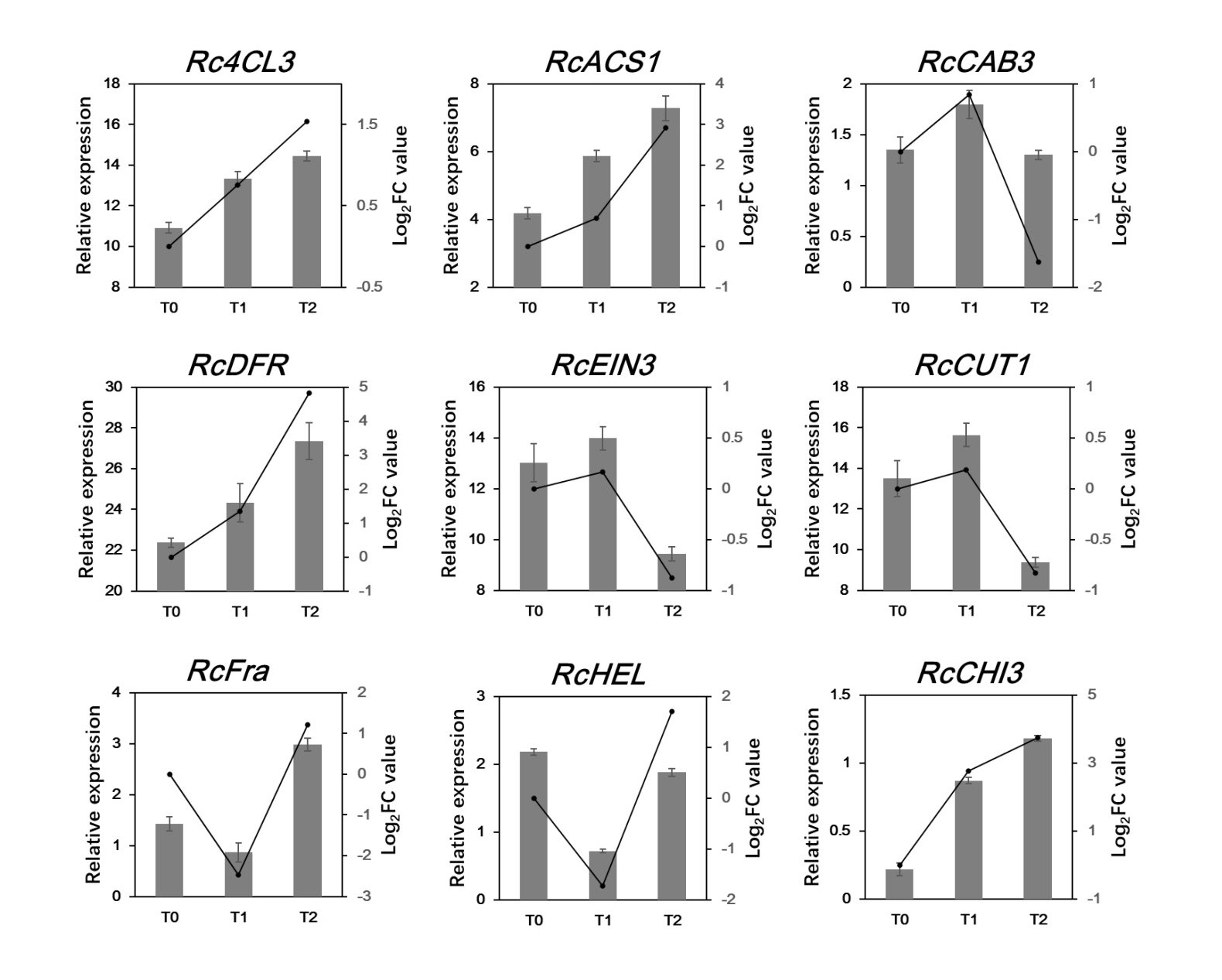

Supplement: Web_Material_uhaf152 [file web_material_uhaf152.zip › Supplementary figure S2.jpg]

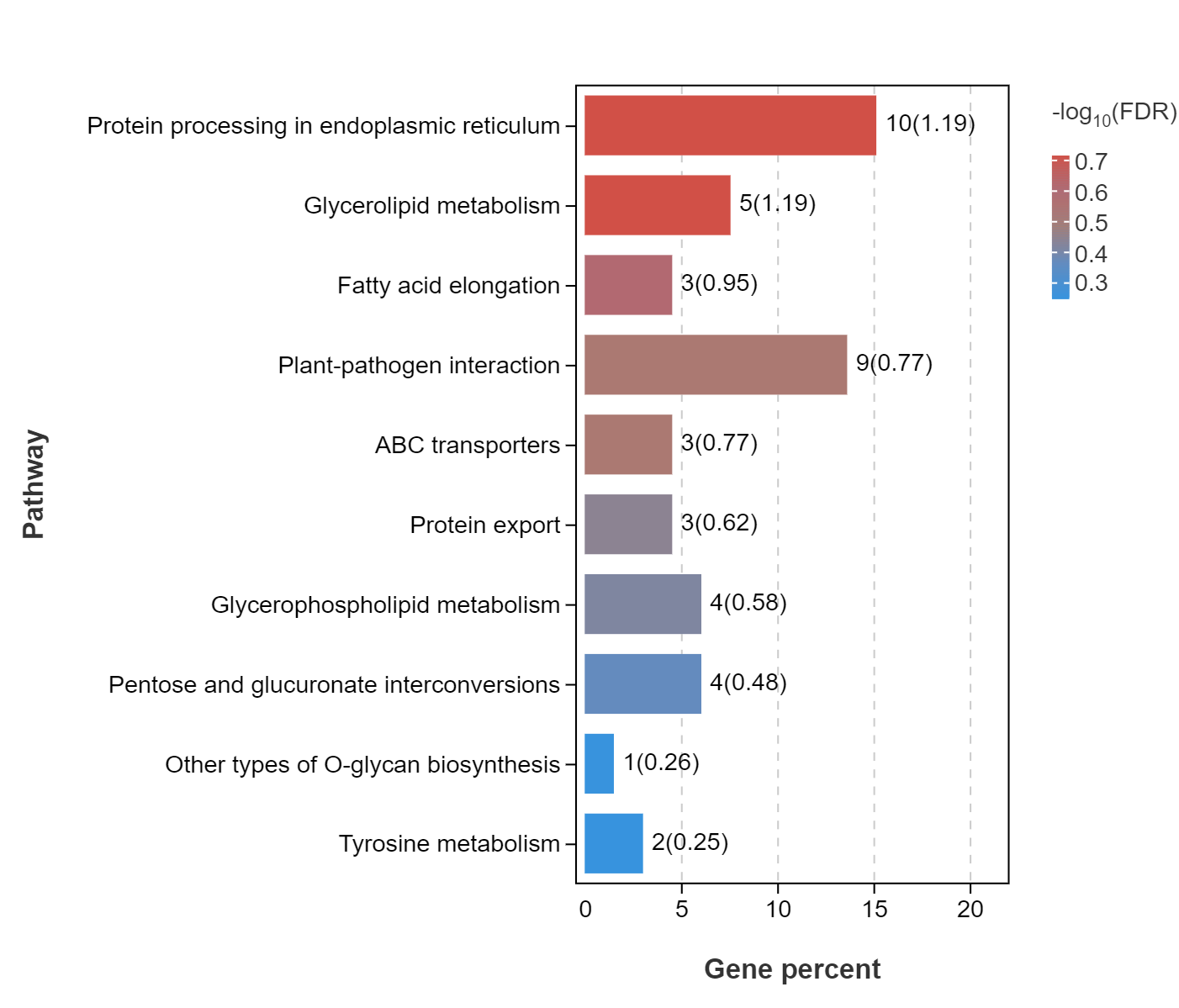

Supplement: Web_Material_uhaf152 [file web_material_uhaf152.zip › Supplementary figure S3.jpeg]

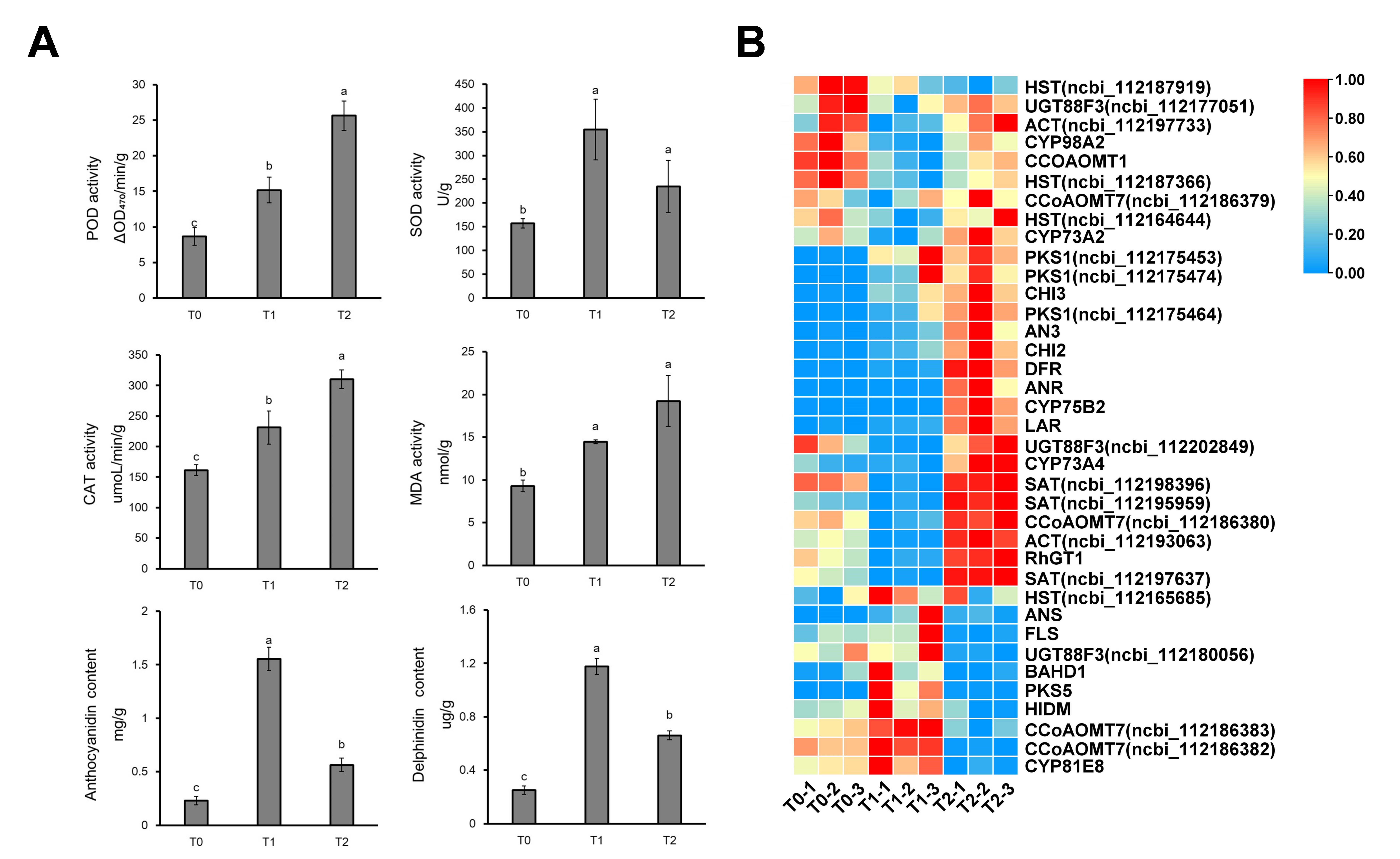

Supplement: Web_Material_uhaf152 [file web_material_uhaf152.zip › Supplementary figure S4.jpg]

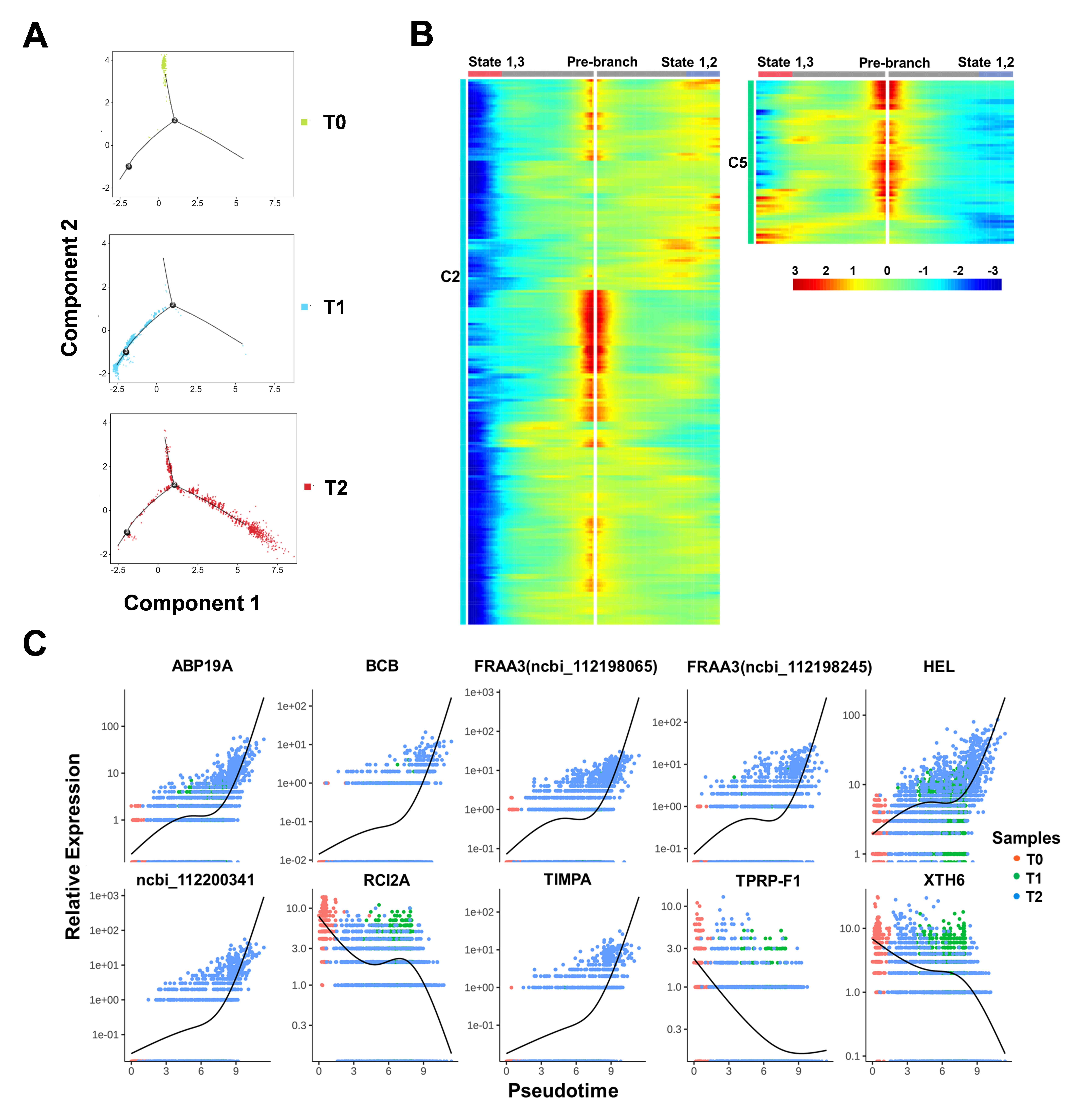

Supplement: Web_Material_uhaf152 [file web_material_uhaf152.zip › Supplementary figure S5.jpg]

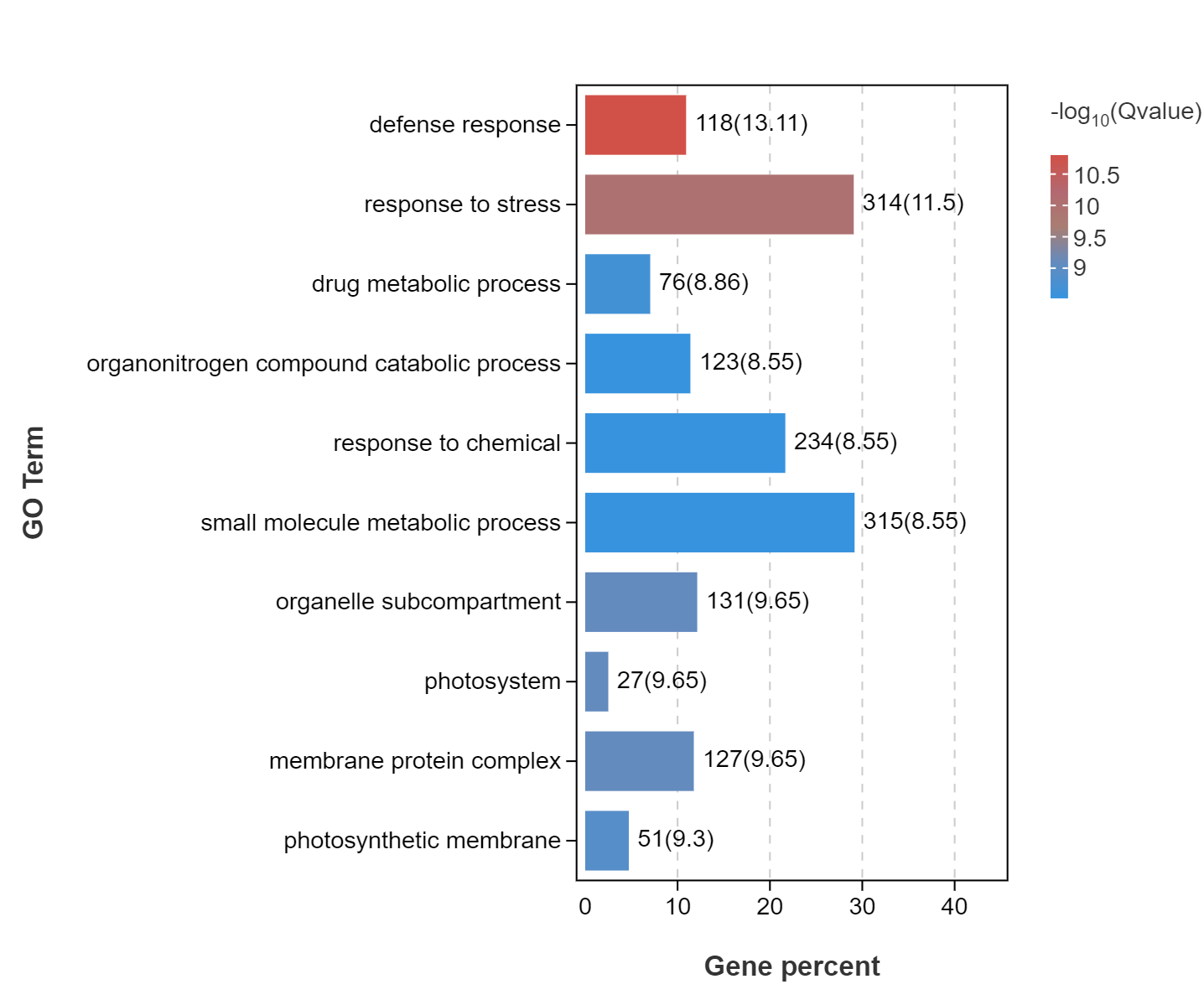

Supplement: Web_Material_uhaf152 [file web_material_uhaf152.zip › Supplementary figure S6.jpeg]

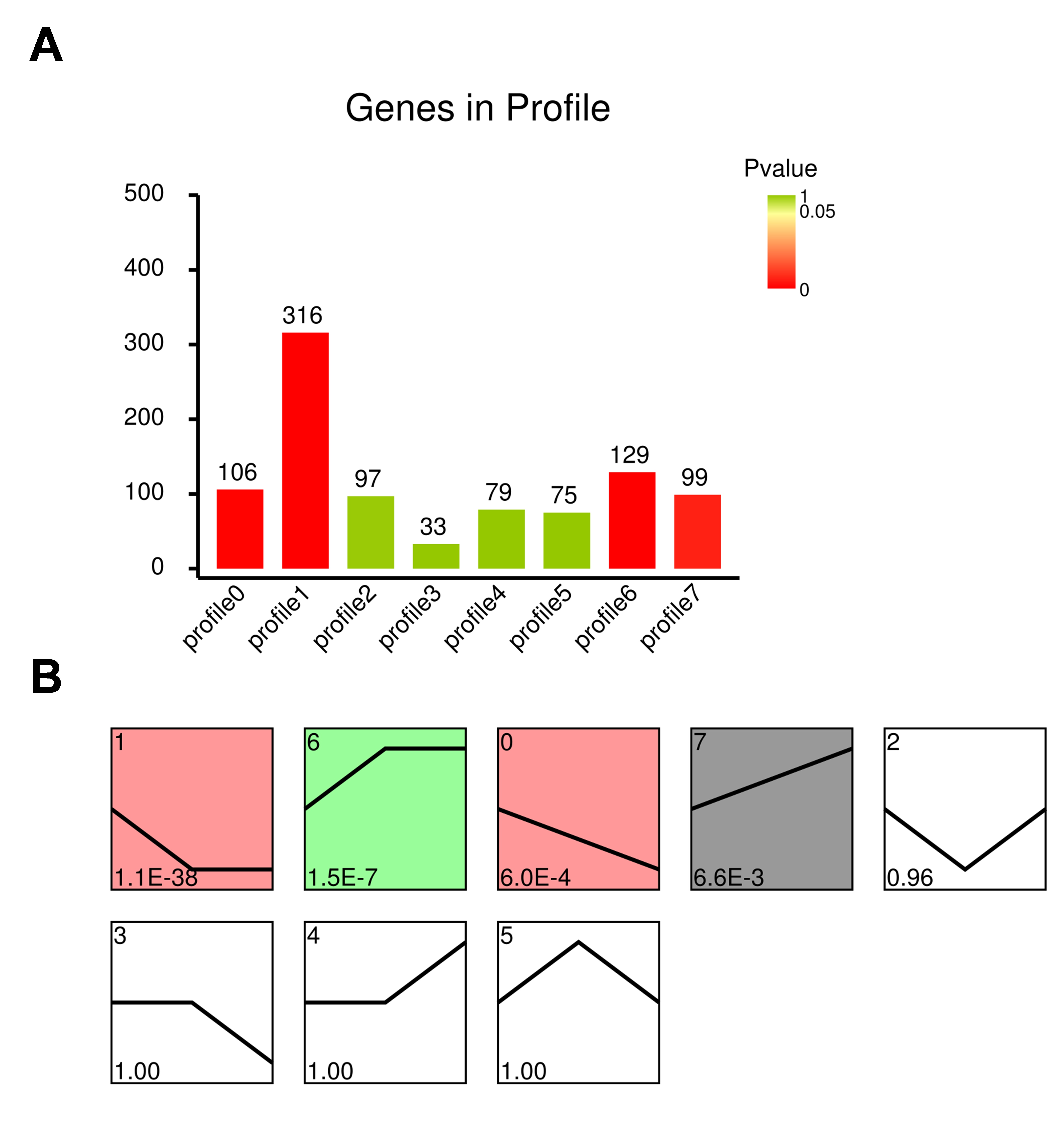

Supplement: Web_Material_uhaf152 [file web_material_uhaf152.zip › Supplementary figure S7.jpg]
